# Supplementary material for: Development and Evaluation of a School Readiness Curriculum for Pediatrics Residents
Source: MedEdPORTAL. 2020 Sep 29;16:10976. doi: 10.15766/mep_2374-8265.10976 (PMC7526503; doi:10.15766/mep_2374-8265.10976)
Supplement: Supplementary file 1 — Preschool Observation Guide.docSchool Readiness Workshop.pptxDevelopmental Questionnaire.pdfPreintervention Survey.docxImmediate Postintervention Survey.docxDelayed Postintervention Survey.docx [file mep_2374-8265.10976-s001.zip › F. Delayed Postintervention Survey.docx]

**School Readiness Curriculum Delayed-Postintervention Survey**

1. In the time since you completed your Developmental-Behavioral Pediatrics rotation, have you received additional formal instruction in school readiness?
2. No
3. Yes
4. In the time since you attended the workshop on school readiness during your Developmental-Behavioral Pediatrics rotation, have you seen a 4- or 5-year-old child for a Well Child Check (WCC)?
   1. No
   2. Yes

If you answered “Yes” to Question 3, please answer the following questions about your most recent Well Child Check (WCC) with a 4- or 5-year-old child:

1. Did **you** bring up the topic of school readiness?
   1. No
   2. Yes
2. Did **the family** bring up the topic of school readiness?
   1. No
   2. Yes
3. How prepared did you feel to answer the family’s questions about school readiness?
   1. Not at all prepared
   2. Slightly prepared
   3. Moderately prepared
   4. Very prepared
   5. Extremely prepared
   6. Not applicable (i.e. the family did not have any questions)
