# Supplementary material for: Development and Evaluation of a School Readiness Curriculum for Pediatrics Residents
Source: MedEdPORTAL. 2020 Sep 29;16:10976. doi: 10.15766/mep_2374-8265.10976 (PMC7526503; doi:10.15766/mep_2374-8265.10976)
Supplement: Supplementary file 1 — Preschool Observation Guide.docSchool Readiness Workshop.pptxDevelopmental Questionnaire.pdfPreintervention Survey.docxImmediate Postintervention Survey.docxDelayed Postintervention Survey.docx [file mep_2374-8265.10976-s001.zip › _Educational_Summary_Report_10976.pdf]

MedEdPORTAL® The AAMC Journal of Teaching and Learning Resources

Original Publication Open Access

Development and Evaluation of a School Readiness Curriculum for Pediatrics Residents

Hannah T. Perrin, MD*, Heidi M. Feldman, MD, PhD, Lynne C. Huffman, MD

*Corresponding Author: Hannah.Perrin@ucsf.edu

Abstract

Introduction: The American Academy of Pediatrics (AAP) recommends that pediatricians promote school readiness with children and families. To our knowledge, no published resident-focused curricula addressing school readiness are currently available. We sought to fill this gap by developing and evaluating a school readiness curriculum for pediatrics residents. **Methods:** We conducted a literature review and targeted needs assessment of pediatrics residents. We then developed a school readiness curriculum and piloted it over several months, adjusting it iteratively each month. The final curriculum was delivered to 34 primarily first-year pediatrics residents over 11 months and included three self-guided observations at local preschools using a templated observation guide, followed by a 1.5-hour in-person facilitated workshop with three components: a PowerPoint presentation, a discussion about preschool observations, and a case study with hands-on developmental questionnaire practice. The curriculum was evaluated with preintervention, immediate postintervention, and 2-months delayed postintervention surveys. **Results:** Our curriculum successfully increased pediatrics residents' knowledge regarding the correct definition of school readiness and appropriate management plan for school readiness concerns, confidence discussing school readiness and addressing families' school readiness concerns, and behavior raising the topic of school readiness with families during well child checks. **Discussion:** A school readiness curriculum had a beneficial effect of increasing pediatrics residents' knowledge, confidence, and behavior addressing school readiness in clinical encounters, meeting a priority of the AAP.

Keywords
School Readiness, Curriculum Evaluation, Preschool, Developmental-Behavioral Pediatrics, Pediatrics, Curriculum Development, Program Evaluation

Educational Objectives

After completing the school readiness curriculum, learners will be able to:

1. Recognize the components of school readiness.
2. Explain the steps to assess the school readiness of a preschool-age child in a general pediatrics clinic.
3. Formulate a management plan for a child who demonstrates weakness in school readiness skills.

Introduction

School readiness is a framework originally defined by the National Education Goals Panel.[1] The framework integrates consideration of a child's skills and behaviors across five readiness-to-learn domains with family, community, and school supports. Collectively, these factors are necessary to promote academic achievement in all children, regardless of their individual skills. The five readiness-to-learn domains include physical well-being and motor development, social and emotional development, approaches to learning, language development, and cognition and general knowledge. Previous research has demonstrated the predictive capability of the school readiness framework in identifying preschool-age children who are at risk for poor academic achievement.[2,3]

The American Academy of Pediatrics (AAP) adopted this framework as a component of health supervision and the basis for assessing a preschool-age child's risk for poor educational outcomes as the child transitions to kindergarten.[4] The AAP recommends that pediatricians discuss school readiness with patients and families in clinic.[5] In order for pediatricians to fulfill this recommendation, they must understand the construct of school readiness, feel comfortable implementing an assessment

Citation:
Perrin HT, Feldman HM, Huffman LC. Development and evaluation of a school readiness curriculum for pediatrics residents. *MedEdPORTAL*. 2020;16:10976.
https://doi.org/10.15766/mep_2374-8265.10976

Copyright © 2020 Perrin et al. This is an open-access publication distributed under the terms of the Creative Commons Attribution license.

and answering questions, and become knowledgeable about approaches to children who demonstrate weakness in one or more of the five prerequisite domains. The 3-year pediatrics residency is the primary opportunity for formal educational experiences for pediatricians-in-training. However, the pediatrics residents in our program reported that they did not receive any formal instruction on school readiness. After conducting a literature review, we identified other pediatrics resident–focused curricula on pediatrics primary care topics that have been published in *MedEdPORTAL*.[6,7] However, we were unable to find any published resident-focused curricula addressing school readiness.

Thus, the primary objectives of our project were twofold: (1) to develop a school readiness curriculum for pediatrics residents using a targeted needs assessment, literature review, expert interviews, and pilot testing and (2) to establish the effectiveness of the new school readiness curriculum by assessing short-term effects on resident knowledge and confidence and medium-term effects on resident behavior.

We used Kern's six-step approach to curriculum development: (1) problem identification and general needs assessment, (2) needs assessment of targeted learners, (3) goals and objectives, (4) educational strategies, (5) implementation, and (6) evaluation and feedback.[8]

We identified the problem as the current lack of training on school readiness for pediatrics residents at our institution, and our general needs assessment literature review showed no readily available published school readiness curricula for residents. We then conducted a targeted needs assessment with approximately 20 pediatrics residents across all years of residency at the Stanford University School of Medicine. Approximately 25% of the residents were able to identify the correct definition of school readiness on a multiple-choice question, but none (0%) of the residents responded correctly to a multiple-choice question about the next step in management when concerned about a child's school readiness. All residents responded that they thought it was extremely important for general pediatricians to be able to assess school readiness in a child, but 50% were not at all confident or only slightly confident in their own abilities to do so. The other 50% were moderately confident, but no residents rated themselves as very confident or extremely confident. Based on the results of the targeted needs assessment, we created educational objectives using Bloom's taxonomy.[9] We aligned our educational strategies with our objectives, using a blended approach of direct instruction and active learning, including experiential site visits to provide residents with hands-on learning. The instructional methods were chosen to maintain congruence with our educational objectives, support a range of learning styles, and be feasible within the constraints of our residency program.[8]

We hope that by sharing this curriculum, other leaders of health training programs will be able to adapt and implement it for their own trainees. Although we targeted pediatrics residents at our institution, this curriculum could be generalized for use with any learners who intend to practice in a primary care setting with young children, including family medicine residents, nurse practitioner students, and physician assistant students.

Methods

Curriculum Development and Delivery

We developed a 1.5-hour workshop on school readiness to address our educational objectives. This didactic experience was designed to complement and expand the existing curriculum, which included three preschool visits that residents completed during their required rotation in developmental-behavioral pediatrics (DBP). The visits were designed originally to enhance appreciation of developmental milestones in preschool-age children. We piloted this workshop with first-year pediatrics residents during their DBP rotations over a period of 4 months in the 2017-2018 academic year. We collected formative evaluations and adjusted the workshop iteratively based on the evaluation results. We delivered the final curriculum, including the three preschool observations and the 1.5-hour workshop, to primarily first-year pediatrics residents during their 1-month DBP rotations over 11 months within one academic year (2018-2019).

Curriculum

During their DBP rotation, pediatrics residents participated in three visits to preschools in the local area: (1) a Head Start program, (2) an inclusion preschool (which integrated children who were developing typically with children who had developmental and/or behavioral conditions), and (3) a private preschool. At each preschool, residents were provided with an observation guide (Appendix A) and were asked to pay particular attention to one child's developmental skills and behaviors. The observation guide was adapted by our institution from an existing observation guide[10] and was designed to help residents learn about the development of young children of various ages. In addition to focusing on the developmental skills of one child, residents were also asked to observe and take notes on the classroom environment, as well as the staff and other children. At the end of the 1-month DBP rotation and after completing

Copyright © 2020 Perrin et al. This is an open-access publication distributed under the terms of the Creative Commons Attribution license.

the preschool observations, residents attended a PowerPoint workshop presentation (Appendix B) facilitated by a third-year fellow in DBP.

This workshop was conducted in person. On average, there were three to four residents at each workshop. The number of residents fluctuated slightly based on the number of residents on the DBP rotation and whether any residents were absent on the day of the workshop. The workshop was scheduled to minimize the number of residents who would be absent due to being postcall or having other service obligations. The location of the workshop varied depending on the residents' schedules on the given day; it occurred either in a private clinic workroom or an office. Residents typically sat in a semicircle, with the facilitator completing the circle.

During the workshop, we provided an overview of school readiness, described current research on school readiness, made recommendations for how to screen for school readiness in primary care clinics, and enumerated evidence-based interventions to address school readiness concerns. Residents participated in a discussion about their preschool visits and were encouraged to use the opportunity to reflect on their observations. As a group, they considered the different educational models for the preschools. Residents then each described a child they had observed using their preschool observation guide and applied their new knowledge about school readiness to evaluate the readiness skills of each child. At the end of the workshop, we engaged residents in a case study with hands-on practice scoring a developmental questionnaire (Ages and Stages Questionnaire [ASQ][11]; Appendix C) and creating a management plan for the child described in the case.

No additional prerequisite knowledge was necessary for the learners. Prerequisite facilitator knowledge included how to score and interpret the developmental questionnaire and an understanding of each of the educational models from the preschool observation visits. Materials for the curriculum included the preschool observation guide (one per preschool visit per resident, which could be delivered to the residents electronically or in paper form prior to the preschool visits), the PowerPoint presentation with computer (and projector if desired), and copies of a sample completed ASQ for practice scoring during the case study (one per resident). The facilitator also had a prescored copy of the ASQ to facilitate the case study discussion. For our project, the facilitator was a third-year fellow in DBP. However, the workshop could be facilitated by a general pediatrician or other instructor of clinical trainees.

Curriculum Evaluation

This project was determined not to meet the definition of human subjects research by the Stanford University Institutional Review Board. Residents completed the preintervention survey (Appendix D) with an attached consent letter at the beginning of the 1-month required rotation. They completed the immediate postintervention survey (Appendix E) right after completion of the workshop. Both surveys assessed resident knowledge and (using a 5-point scale from *not at all* to *extremely confident*) confidence regarding school readiness evaluation and management. The preintervention survey also assessed current resident behavior (i.e., resident clinician discussed school readiness at a recent 4- or 5-year-old well child check [WCC]) and family behavior (i.e., family asked about school readiness at a recent 4- or 5-year-old WCC). A delayed postintervention survey (Appendix F) was administered approximately 2 months after the rotation. We assessed the same resident behavior and family behavior as in the preintervention survey. The preintervention and immediate postintervention surveys were administered on paper because we had in-person direct contact with the residents during their rotation. The delayed postintervention survey was delivered electronically using Research Electronic Data Capture (REDCap)[12] 2 months after completing the DBP rotation because we reasoned that the residents would be scattered among several rotations, precluding a direct contact. The surveys were developed in consultation with experts in the field of medical education research at our institution.

Data Storage

Residents created unique identification codes that were used to link data from the preintervention, immediate postintervention, and delayed postintervention surveys for each participant. Data from the preintervention and immediate postintervention surveys were transcribed into the SPSS software platform and rechecked for accuracy. Similarly, data from the delayed postintervention surveys were stored in REDCap and imported into SPSS.

Data Analysis

We analyzed results using chi-squares for comparing group proportions of dichotomous variables and McNemar's test[13] (for dichotomous variables) and general linear models (for scale variables) to measure changes within participants from preintervention survey baseline.

Results

Participants

Thirty-nine primarily first-year pediatrics residents completed preintervention surveys. Thirty-four residents received the

Copyright © 2020 Perrin et al. This is an open-access publication distributed under the terms of the Creative Commons Attribution license.

workshop and completed immediate postintervention surveys. Three of the 34 residents who completed the workshop and immediate postintervention survey had not completed a preintervention survey, which left 31 residents who completed both preintervention and immediate postintervention surveys. Based on receipt of the workshop and timing of rotation completion, 24 residents were eligible to complete the delayed postintervention survey. This survey was emailed to them approximately 2 months after the rotation; 20 of 24 surveys were returned.

Knowledge

The proportion of resident participants who correctly defined school readiness increased significantly from 28% preintervention (11 of 39 total respondents) to 88% immediate postintervention (30 of 34 total respondents), $\chi^2(1) = 26.59$, $p < .001$ (Figure 1). McNemar's test determined that the difference preintervention to immediate postintervention using paired data was statistically significant ($p < .001$). The proportion who correctly identified the most appropriate management plan for school readiness concerns increased from 13% preintervention (five of 39 total respondents) to 59% immediate postintervention (20 of 34 total respondents), $\chi^2(1) = 17.07$, $p < .001$ (Figure 1). McNemar's test determined that the difference preintervention to immediate postintervention using paired data was statistically significant ($p = .001$).

Confidence

On a 5-point scale from *not at all confident* to *extremely confident*, participant confidence discussing school readiness in general with families increased significantly from a mean of 1.9 preintervention ($SE = 0.15$) to 3.3 immediate postintervention ($SE = 0.15$), $p < .001$ (Figure 2). Participant confidence addressing families' specific school readiness concerns also increased significantly from a mean of 1.6 preintervention ($SE = 0.15$) to 3.2 immediate postintervention ($SE = 0.14$), $p < .001$ (Figure 2).

Behavior

The proportion of participants who reported that they had discussed school readiness during a 4- or 5-year-old's WCC increased significantly from 15% preintervention (four of 27 total respondents) to 75% (nine of 12 total respondents) at the time of the delayed postintervention survey, $\chi^2(1) = 26.59$, $p < .001$ (Figure 3). No participants who, at preintervention, had discussed school readiness at their most recent WCC responded on the delayed postintervention survey that they had not discussed the topic. However, only 10 respondents answered the question on both the preintervention and delayed postintervention surveys. McNemar's test determined that the difference from preintervention to delayed postintervention using paired data was not statistically significant. There was no significant difference in the reported proportion of families who asked about school readiness during a WCC preintervention and at delayed postintervention (15% vs. 42%) using chi-squares or McNemar's test (Figure 3).

Preparedness to Answer Questions

We also asked participants about their preparedness to answer families' questions about school readiness on a 5-point scale, from *not at all prepared* to *extremely prepared.* Only two

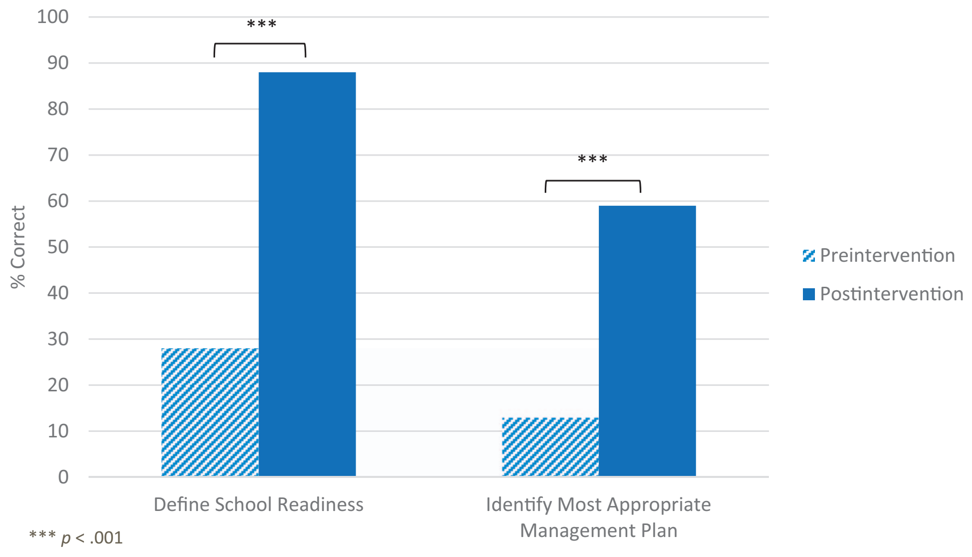

Figure 1. Proportion of residents who correctly defined school readiness preintervention and immediate postintervention (28% vs. 88%) and who correctly identified the most appropriate management plan for a child with school readiness concerns preintervention and immediate postintervention (13% vs. 59%).

Copyright © 2020 Perrin et al. This is an open-access publication distributed under the terms of the Creative Commons Attribution license.

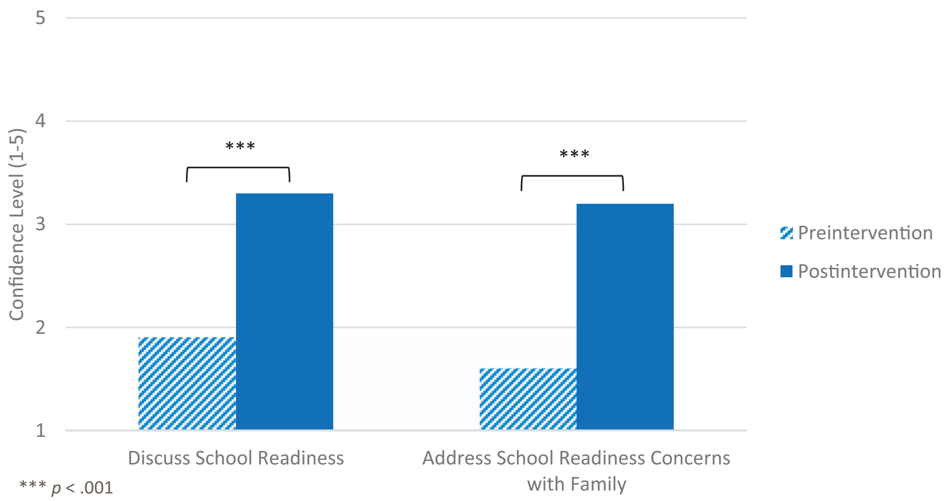

Figure 2. Mean level of confidence discussing school readiness with families on a 5-point scale preintervention and immediate postintervention (1.9 vs. 3.3) and mean level of confidence addressing school readiness concerns with families preintervention and immediate postintervention (1.6 vs. 3.2).

participants reported the family having questions about school readiness both preintervention and at the time of the delayed postintervention survey. The preintervention mean preparedness score was 2.5, and the delayed postintervention mean score was 3.5. These data were not analyzed statistically due to the small number of participants responding to this item.

Resident Evaluation

Residents rated the workshop on one item ("Overall, how would you rate this workshop?") using a 5-point scale (1 = *poor,* 2 = *fair,* 3 = *good,* 4 = *very good,* 5 = *excellent*). The mean score was 4.4 ($SD = 0.7$). All residents rated the workshop between 3 and 5. Qualitative feedback from residents demonstrated an appreciation for the range of instructional methods used. Selected quotes from residents regarding what they enjoyed most about the workshop included the following:

- Knowledge-based/direct instruction:
  - "Going over what school readiness actually means and learning how valuable preschool is."
  - "Reviewing components of school readiness."
  - "Addressing misconceptions surrounding school readiness."
  - "Practical advice re: what to do if not school ready yet."

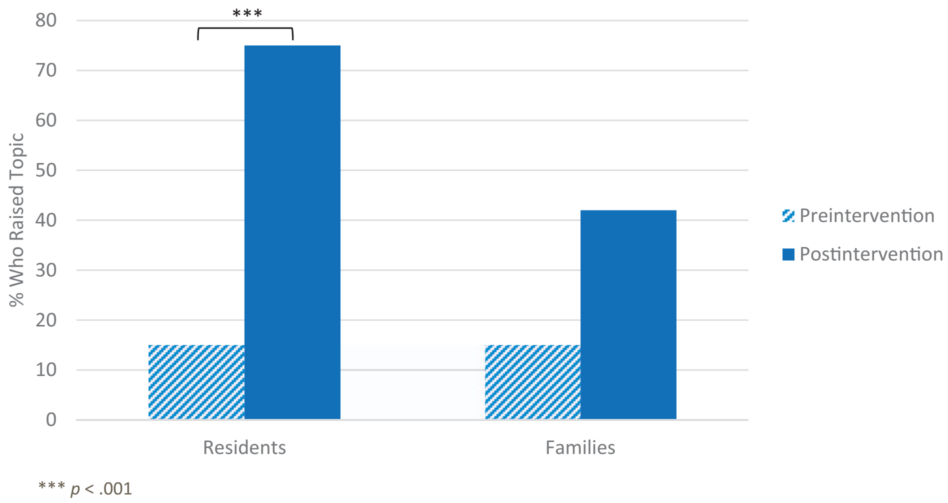

Figure 3. Proportion of residents who raised the topic of school readiness at their most recent 4- or 5-year-old well child check (WCC) preintervention and delayed postintervention (15% vs. 75%) and proportion of residents who reported that families raised the topic of school readiness at their most recent 4- or 5-year-old WCC preintervention and delayed postintervention (15% vs. 42%).

Copyright © 2020 Perrin et al. This is an open-access publication distributed under the terms of the Creative Commons Attribution license.

- Case study/active learning:
  - "Well organized, hands-on, opportunity to participate."
  - "Interactive case and discussion."
  - "Getting to practice scoring the ASQ! Group-based discussion."
  - "Interactive, organized. The case was also a good example of addressing school readiness and applying the principles taught during the discussion."
- Preschool observations/experiential learning:
  - "Specific examples/experiences at preschool observations."
  - "[Appreciated] the fact we all observed at preschools prior to compare what we learned with what we observed."

Discussion

A 1.5-hour workshop with a case study delivered to pediatrics residents during the DBP rotation and combined with three self-guided preschool observations had a beneficial effect of increasing residents' school readiness knowledge, as well as their confidence discussing school readiness and managing school readiness concerns. On a group level, resident behavior in discussing school readiness in clinical encounters also increased following the intervention. However, the change failed to reach clinical significance on an individual participant level due to low numbers of residents who saw a 4- or 5-year-old WCC both preintervention and between the time of the intervention and the delayed postintervention survey. No significant differences were observed in residents' reporting of the family bringing up the topic of school readiness. This finding suggests that we would first see a positive effect on the behavior of the pediatrics residents who received the intervention before we see downstream effects that change behavior among the families under the residents' care. We anticipate that with an increase in numbers of visits during which residents raise the topic, families will also ask questions about school readiness more frequently and that this will eventually become a typical part of every WCC for 4- and 5-year-olds.

The curriculum development process was challenging due to the lack of any published curricula on this topic from which to begin development of a new curriculum. We relied heavily on Kern's six steps[8] and the residents' formative feedback during the pilot phase of the project to guide development. Challenges we encountered during implementation primarily centered around scheduling the preschool observation visits and workshops so that as many residents as possible could attend. This could be a significant limitation for institutions where residents do not have time in their schedules for off-site visits. Although we prioritized having residents visit three preschools with different educational models so that they could become familiar with each model through experiential learning, the curriculum could also be adapted for residents who are able to visit only one or two preschools, which might be more feasible for other institutions. The observation guide and discussion prompts could be easily generalized to any location where residents are able to visit preschools using different educational models. Alternatively, the curriculum could be adapted to teach about preschool educational models and provide examples of preschool-age child development through videos if in-person observations are not feasible. Although our residents spoke highly of the preschool observations, a benefit of video teaching would be the inclusion of a carefully curated shared experience from which to observe a child's development across school readiness domains together and discuss that child in the moment. Additionally, the case study with practice scoring the developmental questionnaire can be adjusted to accommodate another questionnaire that might be utilized more frequently at another institution.

One limitation of our project evaluation was low participant numbers. Our response rate for survey completion was adequate: 100% of residents who received the intervention completed immediate postintervention surveys, and 83% of residents (20 out of 24) who were eligible to complete delayed postintervention surveys did so. However, given the timing of the resident rotations and delayed postintervention surveys, only 24 residents were eligible to complete the delayed postintervention surveys by the end of the project, resulting in fewer delayed postintervention surveys than participants. Furthermore, fewer residents than anticipated had seen a 4- or 5-year-old WCC both preintervention and by the time of the delayed postintervention survey, which resulted in a low number of responses to the behavior and preparedness questions. A second limitation of the project evaluation was that the behavior data were collected using resident self-report. Ideally, we would also have data from the resident's supervisor and/or from patient families. Given that residents were at multiple continuity sites with many different supervisors, this would have been quite challenging for the current project. However, it would certainly be recommended for any future evaluations of this curriculum.

Future directions for this work include implementation in other pediatrics residency programs or other clinical training programs to test generalizability, as well as assessment of longer-term behavioral outcomes in the residents and, eventually, patient outcomes. Our hope is that by disseminating this curriculum to

Copyright © 2020 Perrin et al. This is an open-access publication distributed under the terms of the Creative Commons Attribution license.

a wider population, school readiness will come to be included as part of a standard pediatrics residency curriculum in order to prepare pediatricians to discuss this important topic with patients and families.

Appendices

A. Preschool Observation Guide.doc

B. School Readiness Workshop.pptx

C. Developmental Questionnaire.pdf

D. Preintervention Survey.docx

E. Immediate Postintervention Survey.docx

F. Delayed Postintervention Survey.docx

All appendices are peer reviewed as integral parts of the Original Publication.

Hannah T. Perrin, MD: Assistant Professor, Developmental Medicine, University of California, San Francisco, School of Medicine

Heidi M. Feldman, MD, PhD: Professor, Developmental-Behavioral Pediatrics, Stanford University School of Medicine

Lynne C. Huffman, MD: Associate Professor, Developmental-Behavioral Pediatrics, Stanford University School of Medicine

Disclosures
None to report.

Funding/Support
None to report.

Ethical Approval
Reported as not applicable.

References

1. Copple CE; Goal 1 Early Childhood Assessments Resource Group. *Getting a Good Start in School*. National Education Goals Panel; 1997. https://files.eric.ed.gov/fulltext/ED412025.pdf
2. Pritchard VE, Bora S, Austin NC, Levin KJ, Woodward LJ. Identifying very preterm children at educational risk using a school readiness framework. *Pediatrics*. 2014;134(3):e825-e832. https://doi.org/10.1542/peds.2013-3865
3. Pagani LS, Fitzpatrick C, Archambault I, Janosz M. School readiness and later achievement: a French Canadian replication and extension. *Dev Psychol*. 2010;46(5):984-994. https://doi.org/10.1037/a0018881
4. High PC; Committee on Early Childhood, Adoption, and Dependent Care; Council on School Health. School readiness. *Pediatrics*. 2008;121(4):e1008-e1015. https://doi.org/10.1542/peds.2008-0079
5. Council on Early Childhood; Council on School Health. The pediatrician's role in optimizing school readiness. *Pediatrics*. 2016;138(3):e20162293. https://doi.org/10.1542/peds.2016-2293
6. Essel KD, Yalamanchi S, Hysom E, Lichtenstein C. Healthy homes, healthy futures: a home visitation curriculum for pediatric residents. *MedEdPORTAL*. 2016;12:10480. https://doi.org/10.15766/mep_2374-8265.10480
7. Boykan R, Quinn L, Messina C. GUIDES: assessment for prevention of child maltreatment in primary care. *MedEdPORTAL*. 2013;9:9429. https://doi.org/10.15766/mep_2374-8265.9429
8. Thomas PA, Kern DE, Hughes MT, Chen BY, eds. *Curriculum Development for Medical Education: A Six-Step Approach*. 3rd ed. Johns Hopkins University Press; 2016.
9. Anderson LW, Krathwohl DR, eds. *A Taxonomy for Learning, Teaching, and Assessing: A Revision of Bloom's Taxonomy of Educational Objectives*. Addison Wesley Longman: 2001.
10. Sharp MC, Lorch SC. A community outreach training program for pediatric residents and medical students. *J Med Educ*. 1988;63(4):316-322. https://doi.org/10.1097/00001888-198804000-00008
11. Squires J, Bricker D. *Ages & Stages Questionnaires: A Parent-Completed Child Monitoring System*. Paul H. Brookes Publishing Co; 2009.
12. Harris PA, Taylor R, Thielke R, Payne J, Gonzalez N, Conde JG. Research electronic data capture (REDCap)—a metadata-driven methodology and workflow process for providing translational research informatics support. *J Biomed Inform*. 2009;42(2):377-381. https://doi.org/10.1016/j.jbi.2008.08.010
13. McNemar Q. Note on the sampling error of the difference between correlated proportions or percentages. *Psychometrika*. 1947;12(2):153-157.

Received: October 12, 2019
Accepted: March 27, 2020
Published: September 29, 2020

Copyright © 2020 Perrin et al. This is an open-access publication distributed under the terms of the Creative Commons Attribution license.
