# Supplementary material for: Development and Evaluation of a School Readiness Curriculum for Pediatrics Residents
Source: MedEdPORTAL. 2020 Sep 29;16:10976. doi: 10.15766/mep_2374-8265.10976 (PMC7526503; doi:10.15766/mep_2374-8265.10976)
Supplement: Supplementary file 1 — Preschool Observation Guide.docSchool Readiness Workshop.pptxDevelopmental Questionnaire.pdfPreintervention Survey.docxImmediate Postintervention Survey.docxDelayed Postintervention Survey.docx [file mep_2374-8265.10976-s001.zip › B. School Readiness Workshop.pptx]

## Slide 1
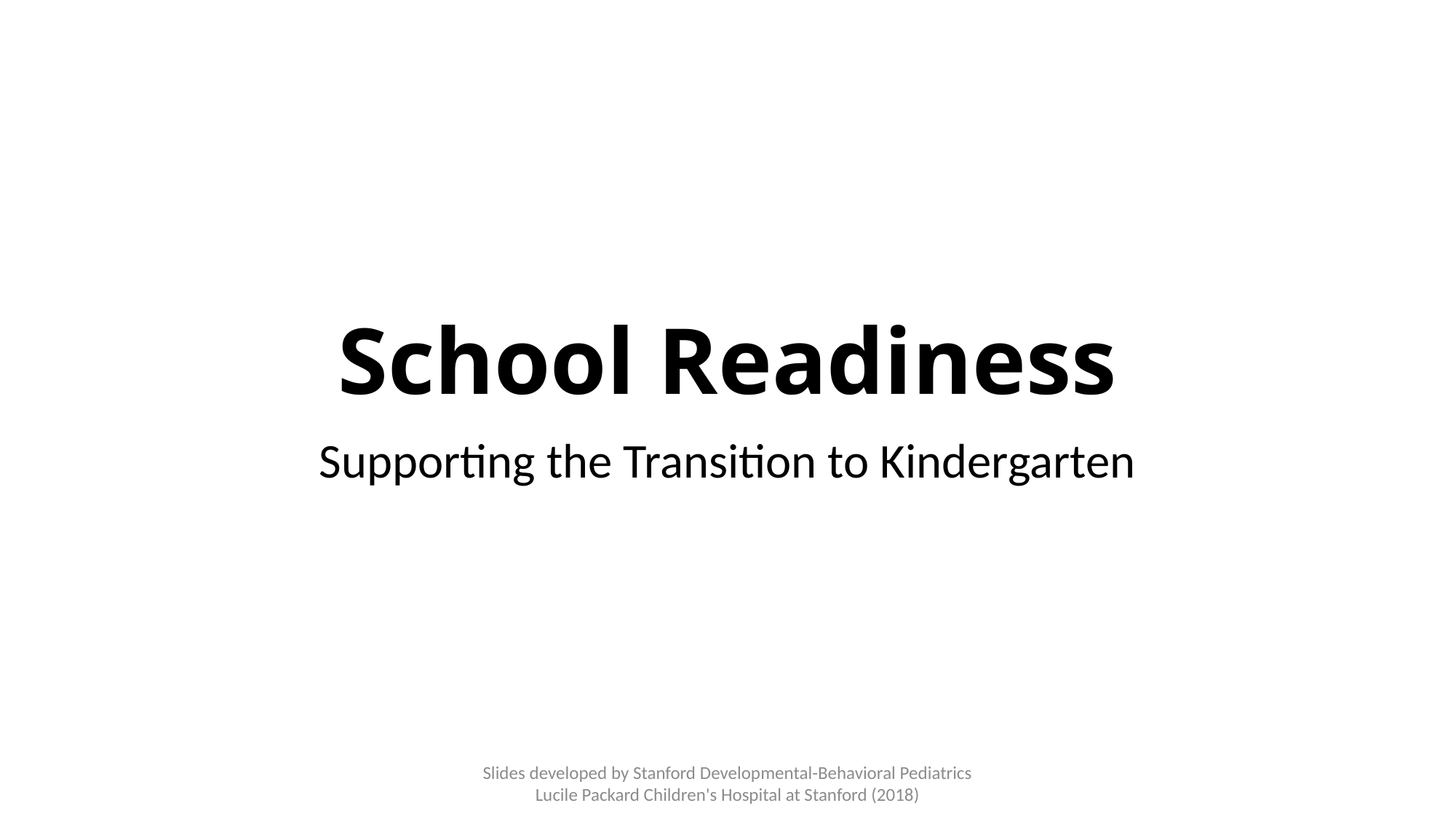

# School Readiness
Supporting the Transition to Kindergarten
Slides developed by Stanford Developmental-Behavioral Pediatrics Lucile Packard Children's Hospital at Stanford (2018)

## Slide 2
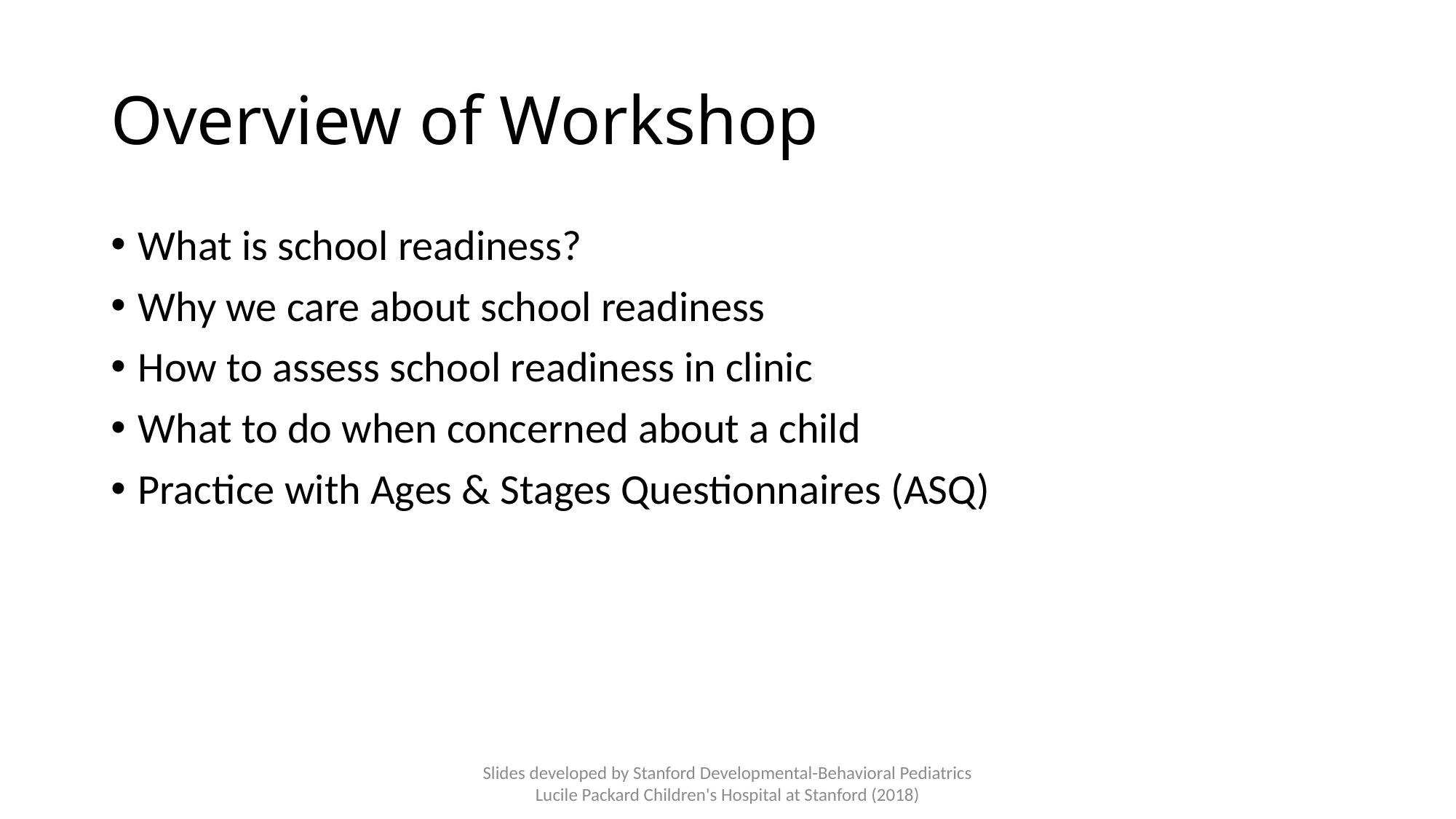

# Overview of Workshop
What is school readiness?
Why we care about school readiness
How to assess school readiness in clinic
What to do when concerned about a child
Practice with Ages & Stages Questionnaires (ASQ)
Slides developed by Stanford Developmental-Behavioral Pediatrics Lucile Packard Children's Hospital at Stanford (2018)

## Slide 3
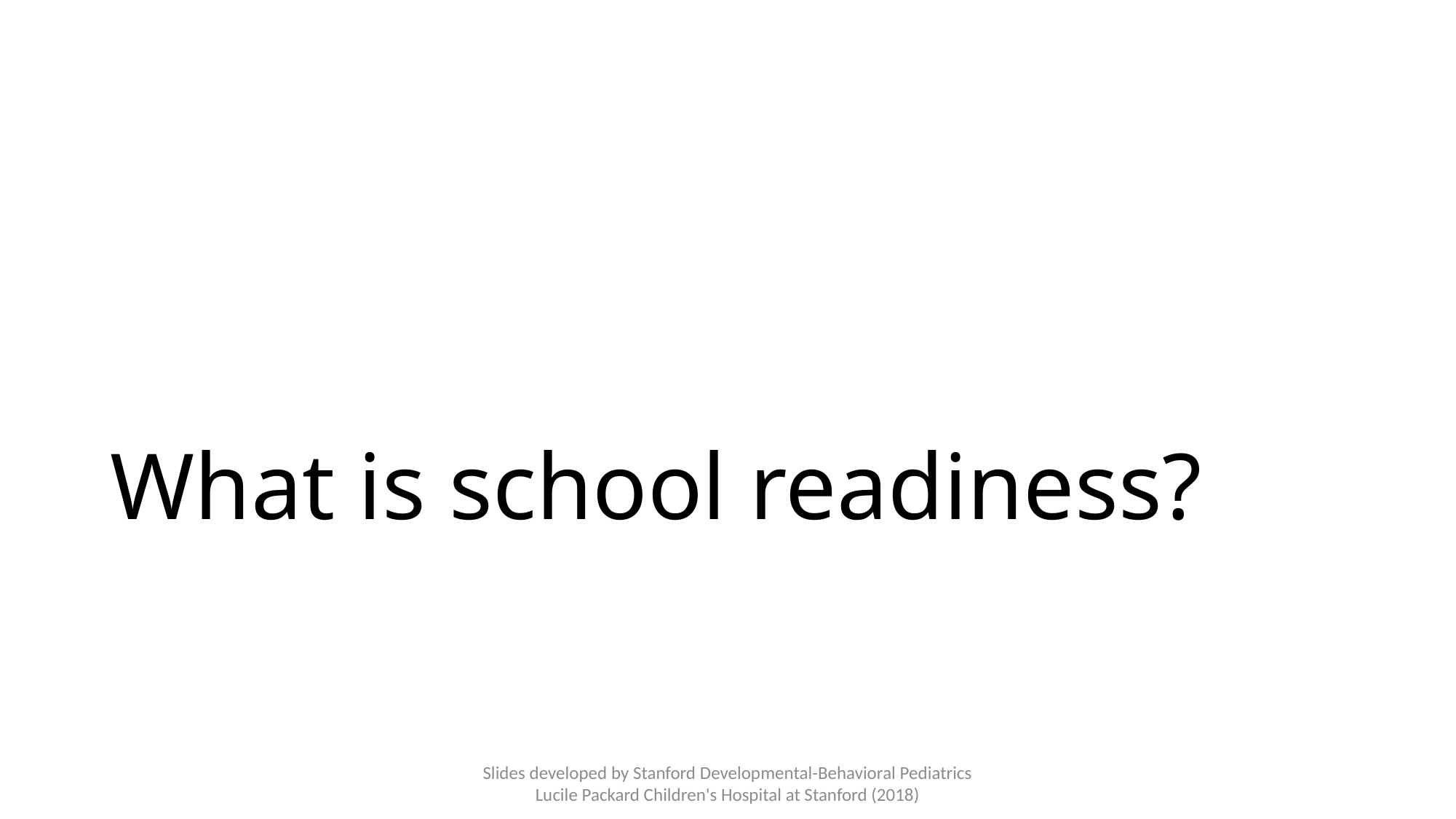

# What is school readiness?
Slides developed by Stanford Developmental-Behavioral Pediatrics Lucile Packard Children's Hospital at Stanford (2018)

## Slide 4
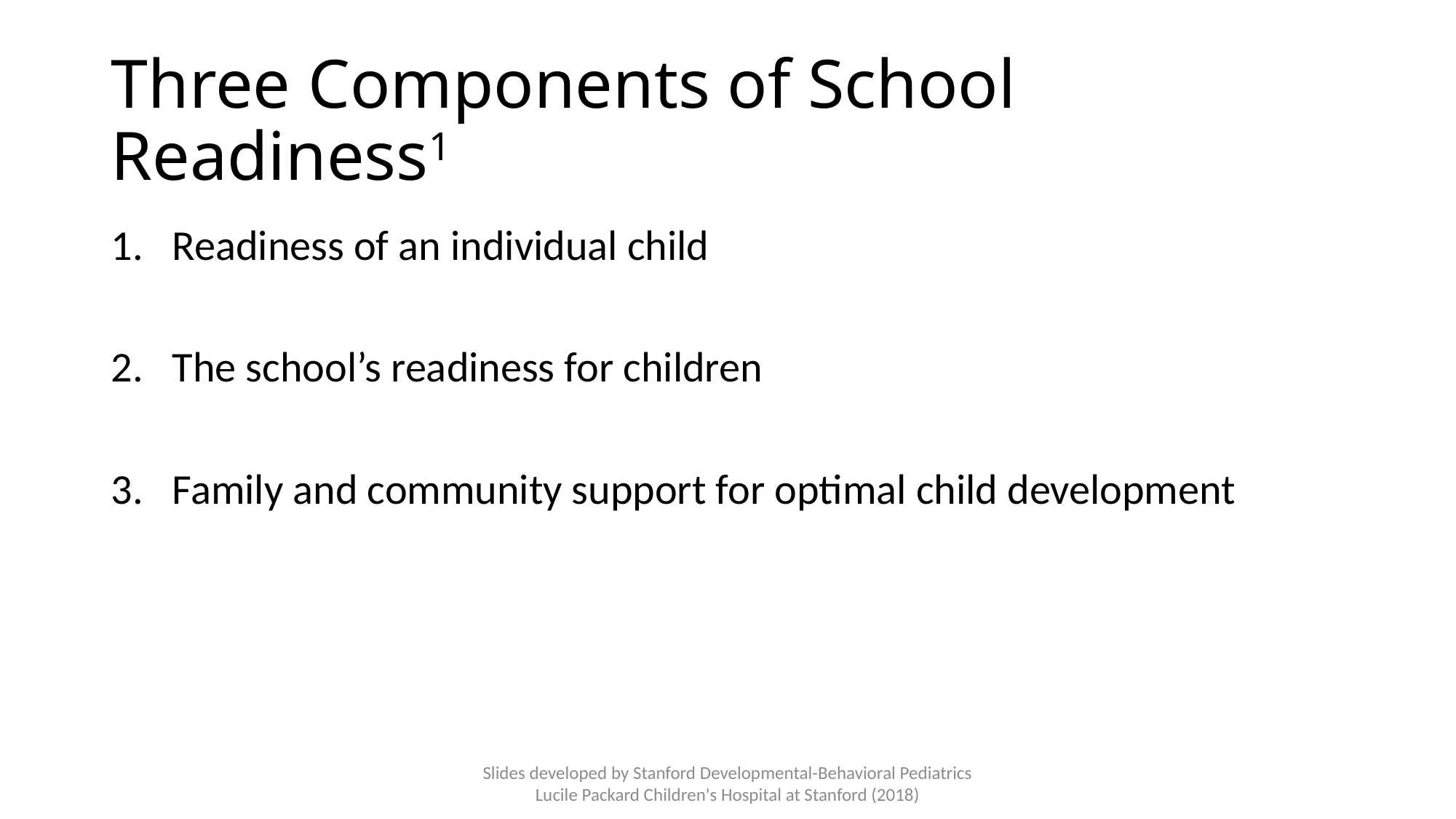

# Three Components of School Readiness1
Readiness of an individual child
The school’s readiness for children
Family and community support for optimal child development
Slides developed by Stanford Developmental-Behavioral Pediatrics Lucile Packard Children's Hospital at Stanford (2018)

## Slide 5
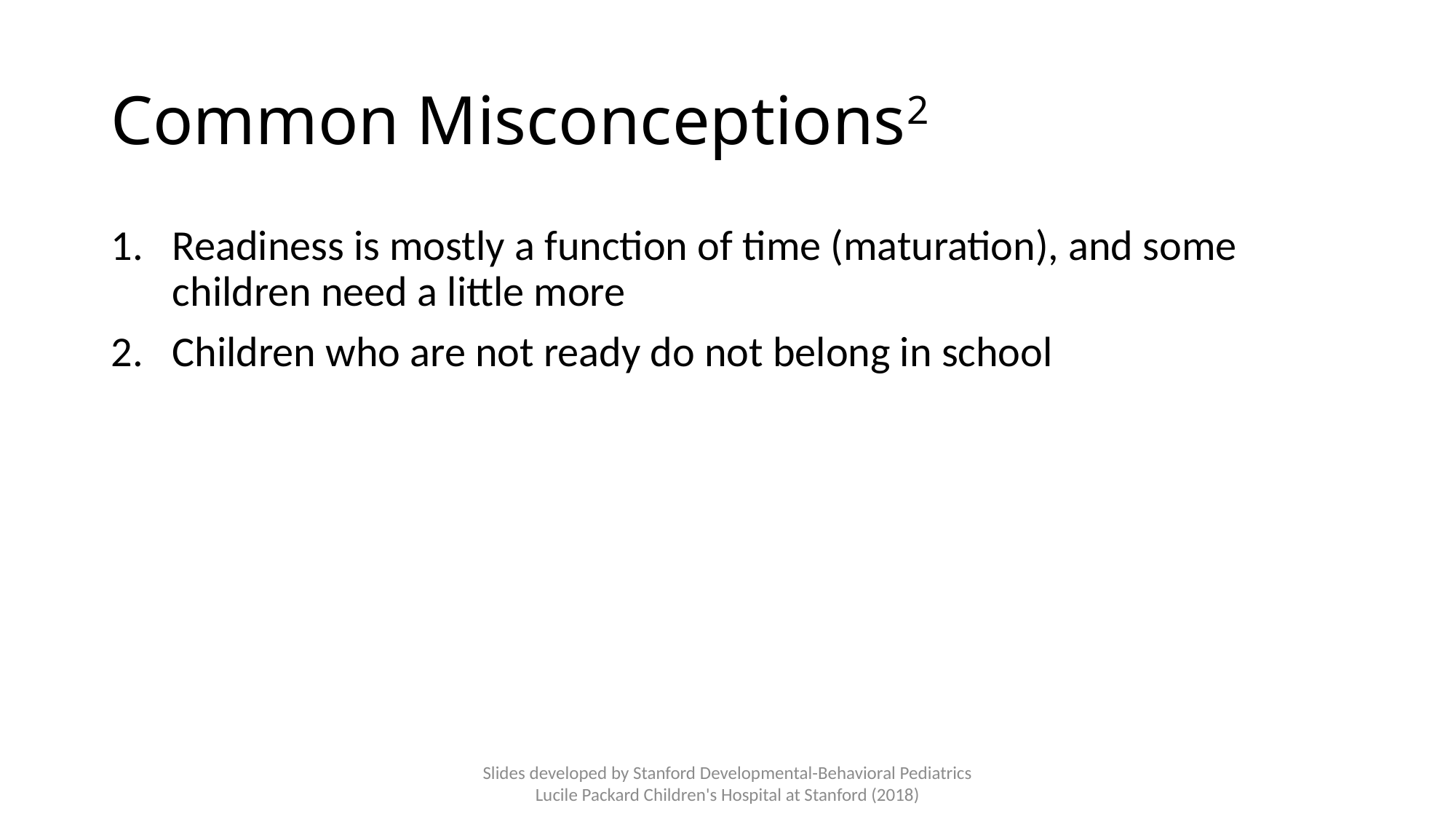

# Common Misconceptions2
Readiness is mostly a function of time (maturation), and some children need a little more
Children who are not ready do not belong in school
Slides developed by Stanford Developmental-Behavioral Pediatrics Lucile Packard Children's Hospital at Stanford (2018)

## Slide 6
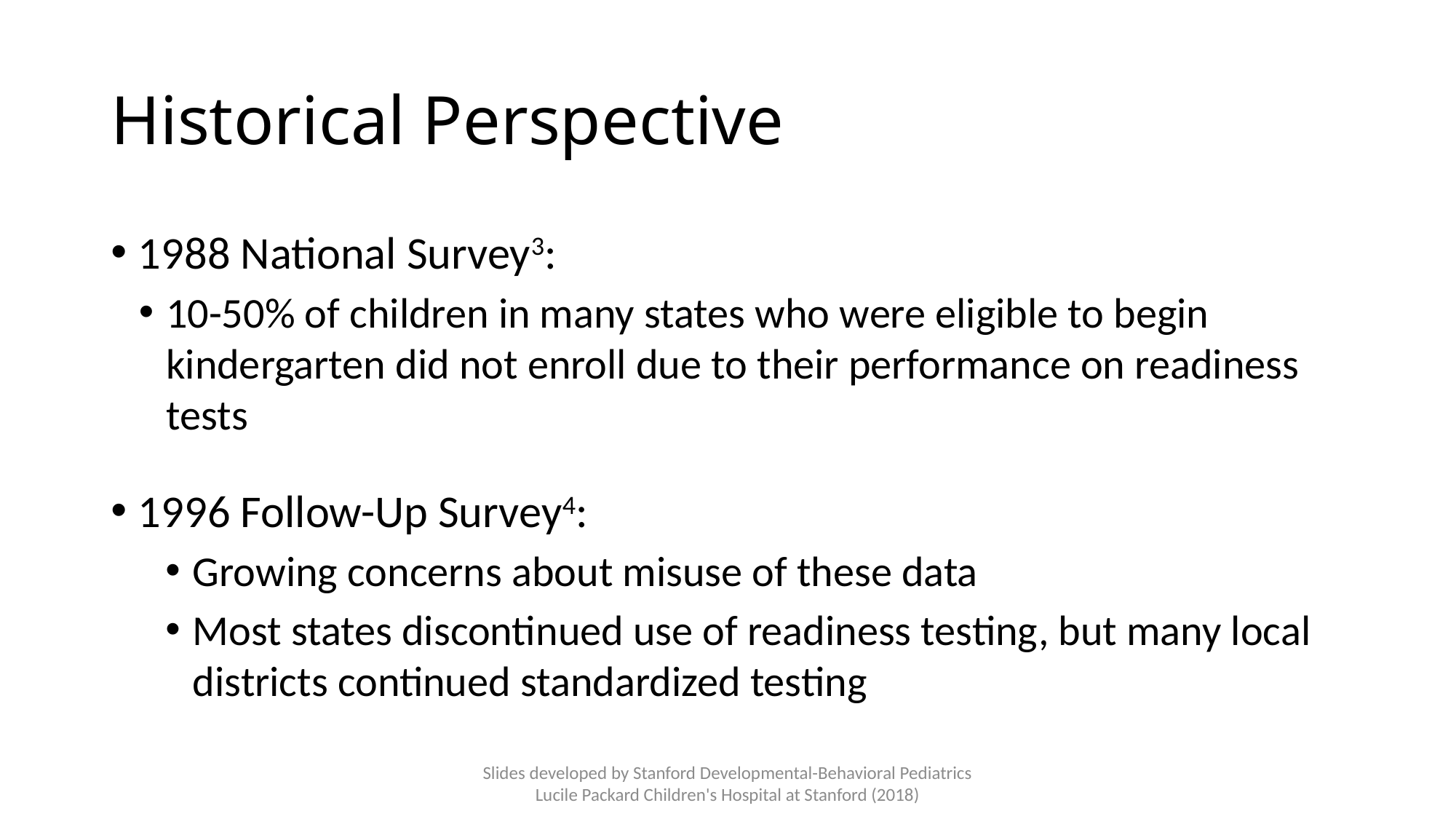

# Historical Perspective
1988 National Survey3:
10-50% of children in many states who were eligible to begin kindergarten did not enroll due to their performance on readiness tests
1996 Follow-Up Survey4:
Growing concerns about misuse of these data
Most states discontinued use of readiness testing, but many local districts continued standardized testing
Slides developed by Stanford Developmental-Behavioral Pediatrics Lucile Packard Children's Hospital at Stanford (2018)

## Slide 7
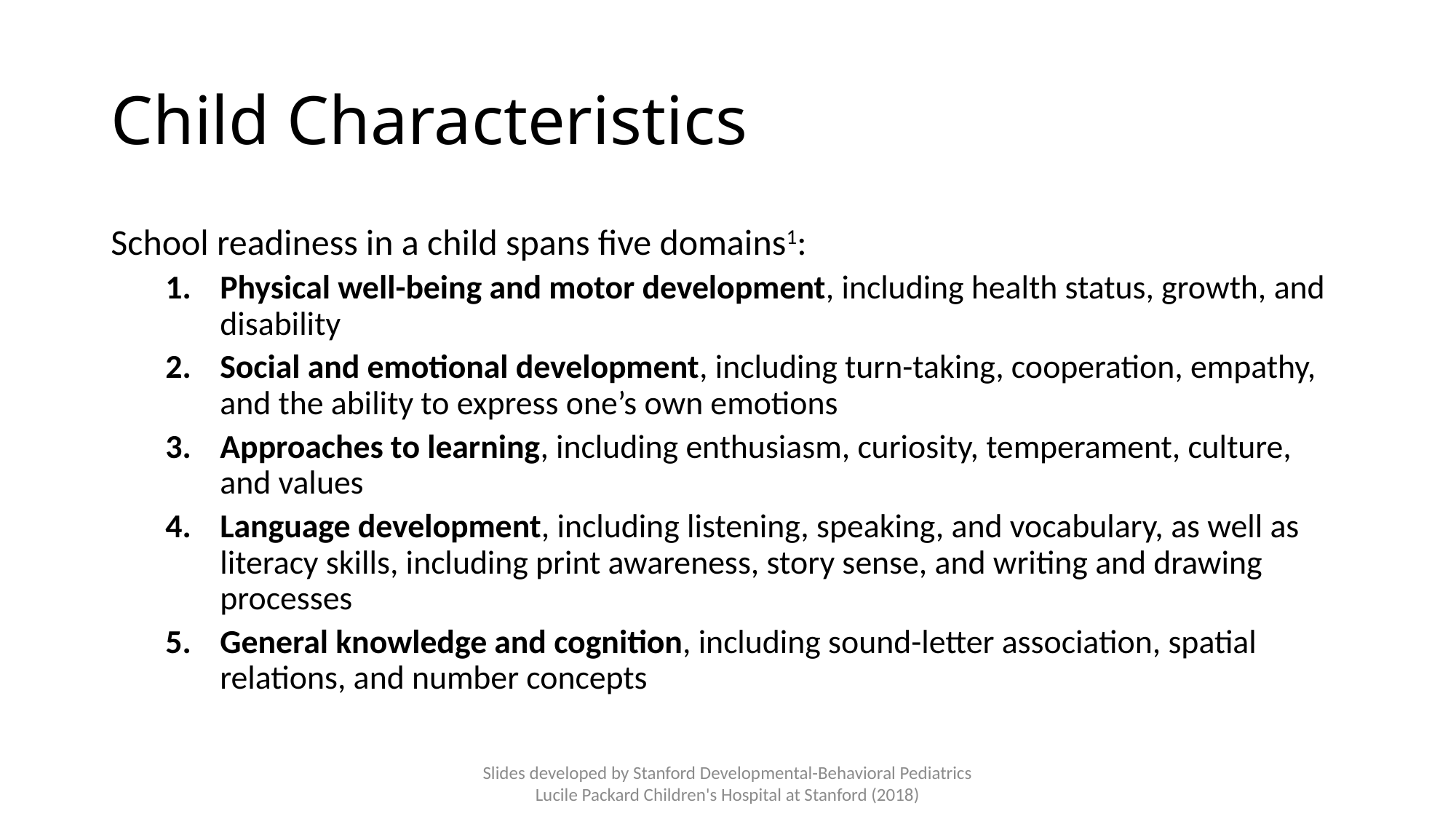

# Child Characteristics
School readiness in a child spans five domains1:
Physical well-being and motor development, including health status, growth, and disability
Social and emotional development, including turn-taking, cooperation, empathy, and the ability to express one’s own emotions
Approaches to learning, including enthusiasm, curiosity, temperament, culture, and values
Language development, including listening, speaking, and vocabulary, as well as literacy skills, including print awareness, story sense, and writing and drawing processes
General knowledge and cognition, including sound-letter association, spatial relations, and number concepts
Slides developed by Stanford Developmental-Behavioral Pediatrics Lucile Packard Children's Hospital at Stanford (2018)

## Slide 8
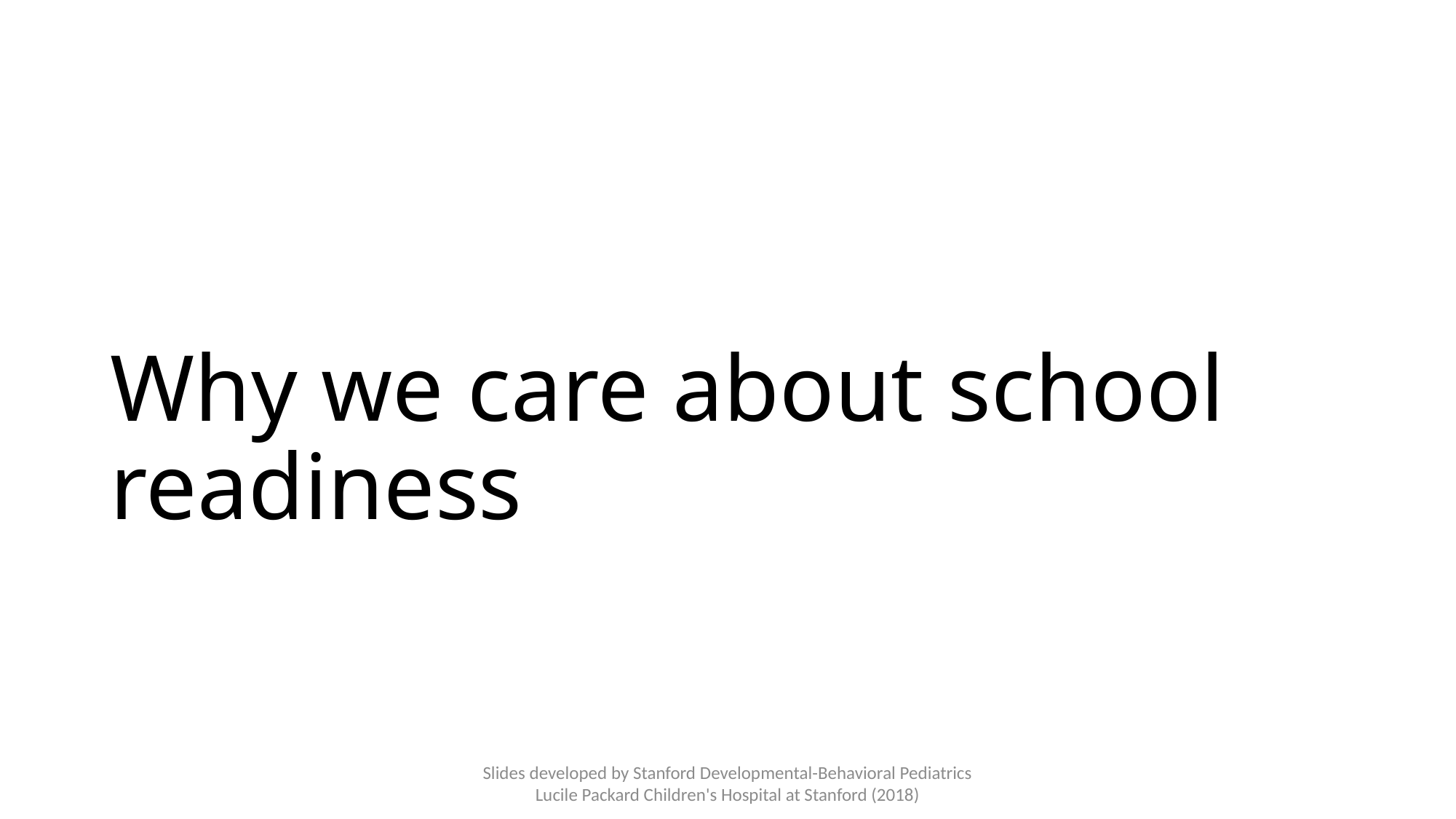

# Why we care about school readiness
Slides developed by Stanford Developmental-Behavioral Pediatrics Lucile Packard Children's Hospital at Stanford (2018)

## Slide 9
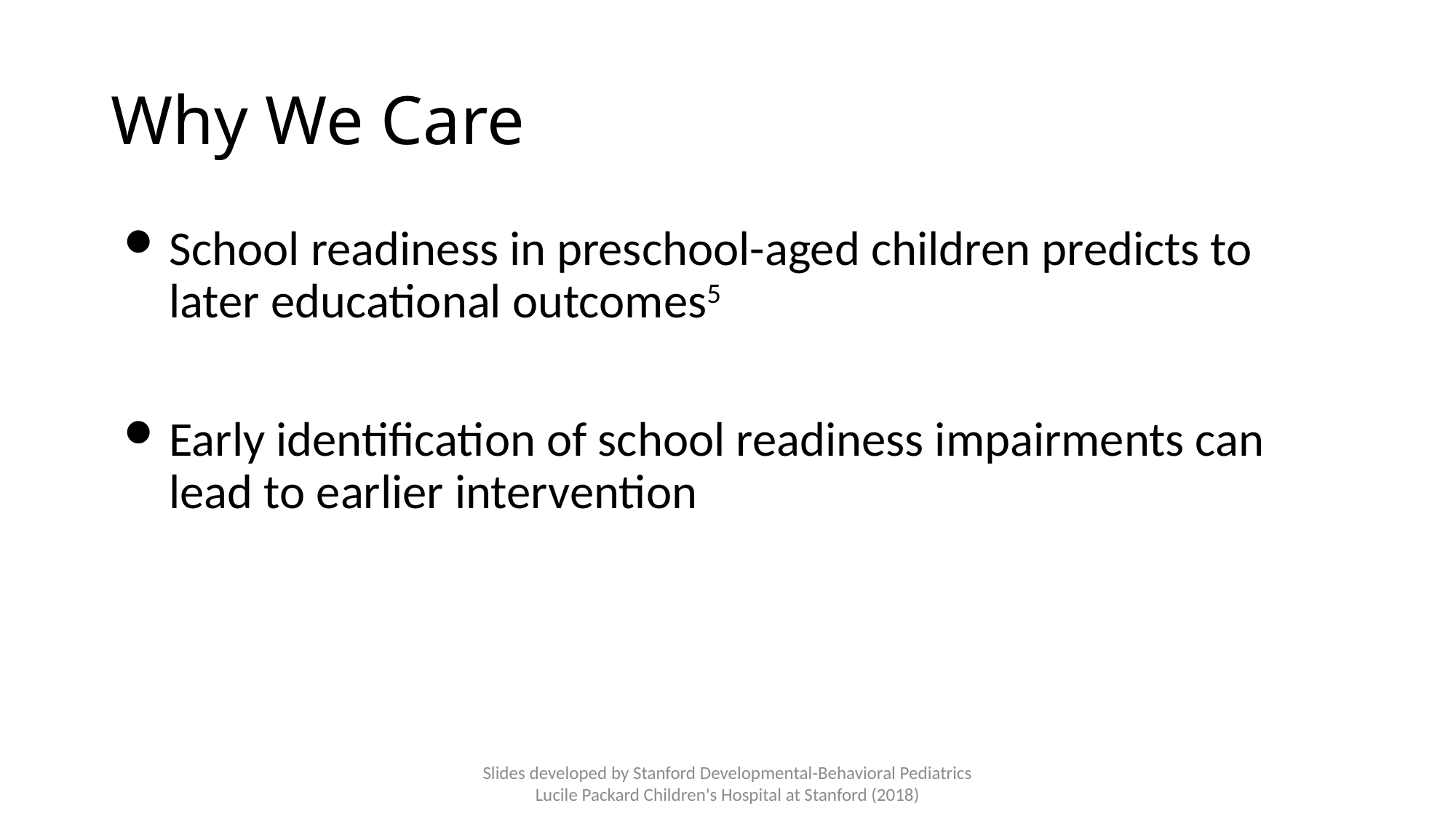

# Why We Care
School readiness in preschool-aged children predicts to later educational outcomes5
Early identification of school readiness impairments can lead to earlier intervention
Slides developed by Stanford Developmental-Behavioral Pediatrics Lucile Packard Children's Hospital at Stanford (2018)

## Slide 10
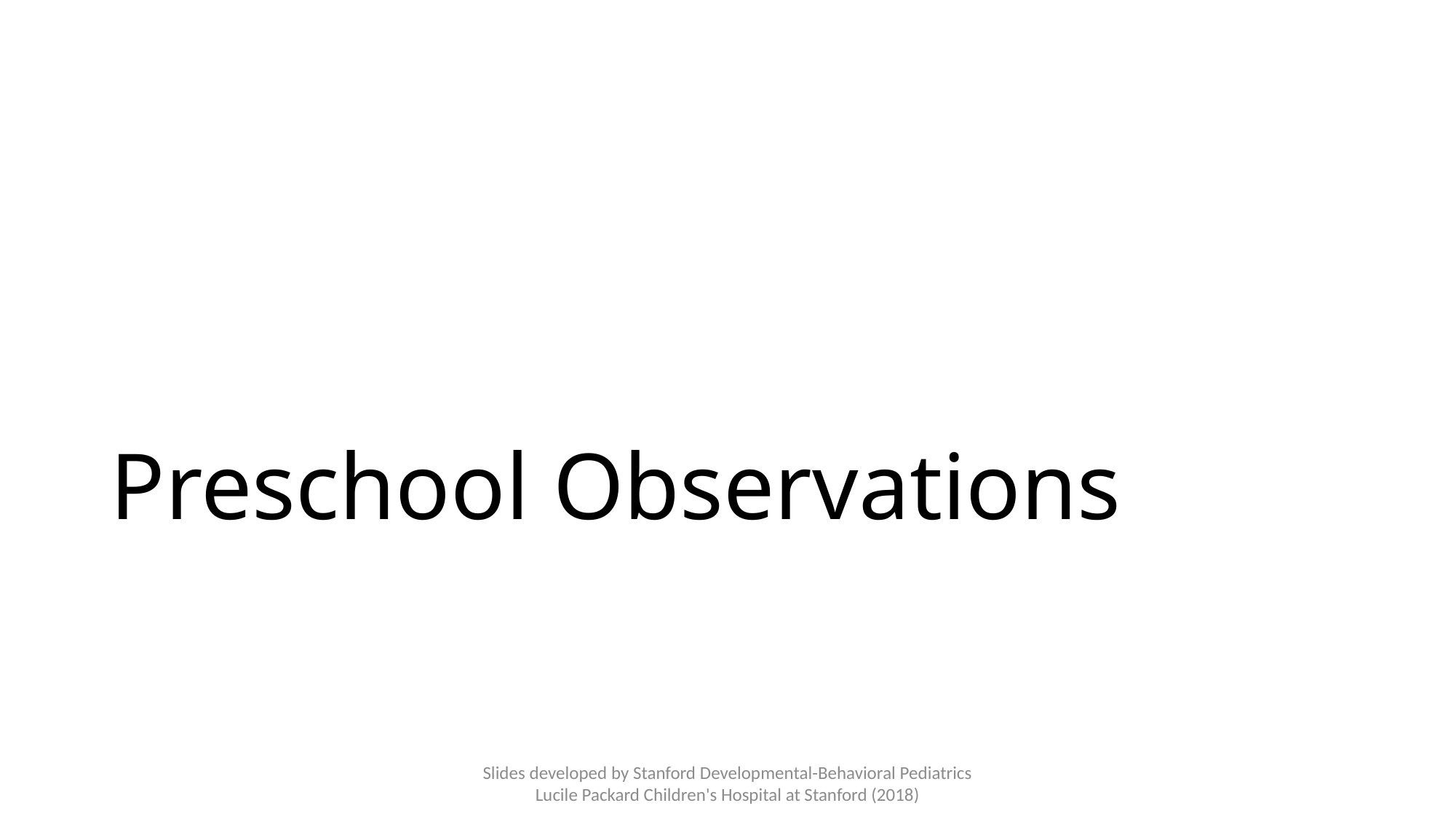

# Preschool Observations
Slides developed by Stanford Developmental-Behavioral Pediatrics Lucile Packard Children's Hospital at Stanford (2018)

## Slide 11
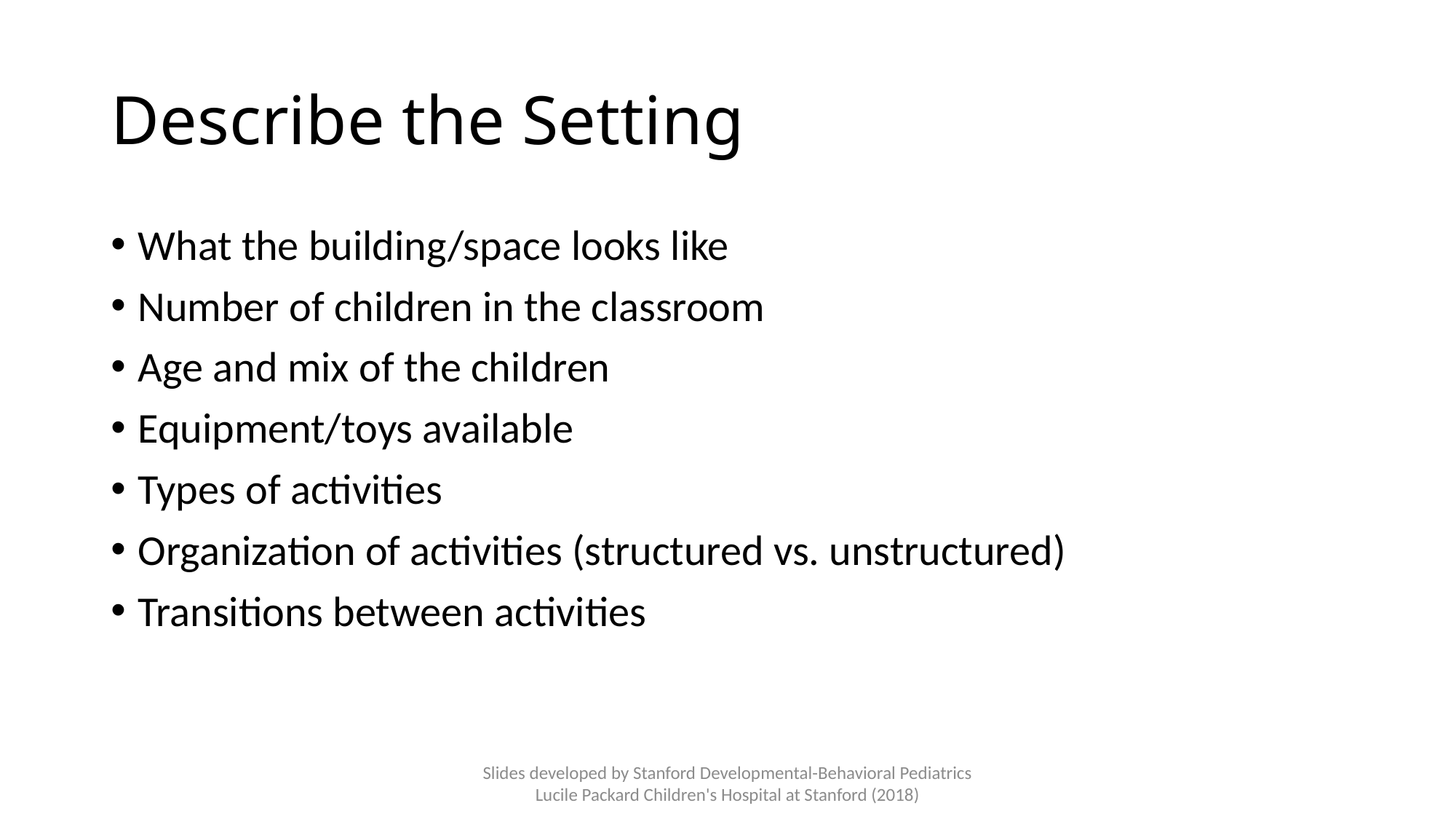

# Describe the Setting
What the building/space looks like
Number of children in the classroom
Age and mix of the children
Equipment/toys available
Types of activities
Organization of activities (structured vs. unstructured)
Transitions between activities
Slides developed by Stanford Developmental-Behavioral Pediatrics Lucile Packard Children's Hospital at Stanford (2018)

## Slide 12
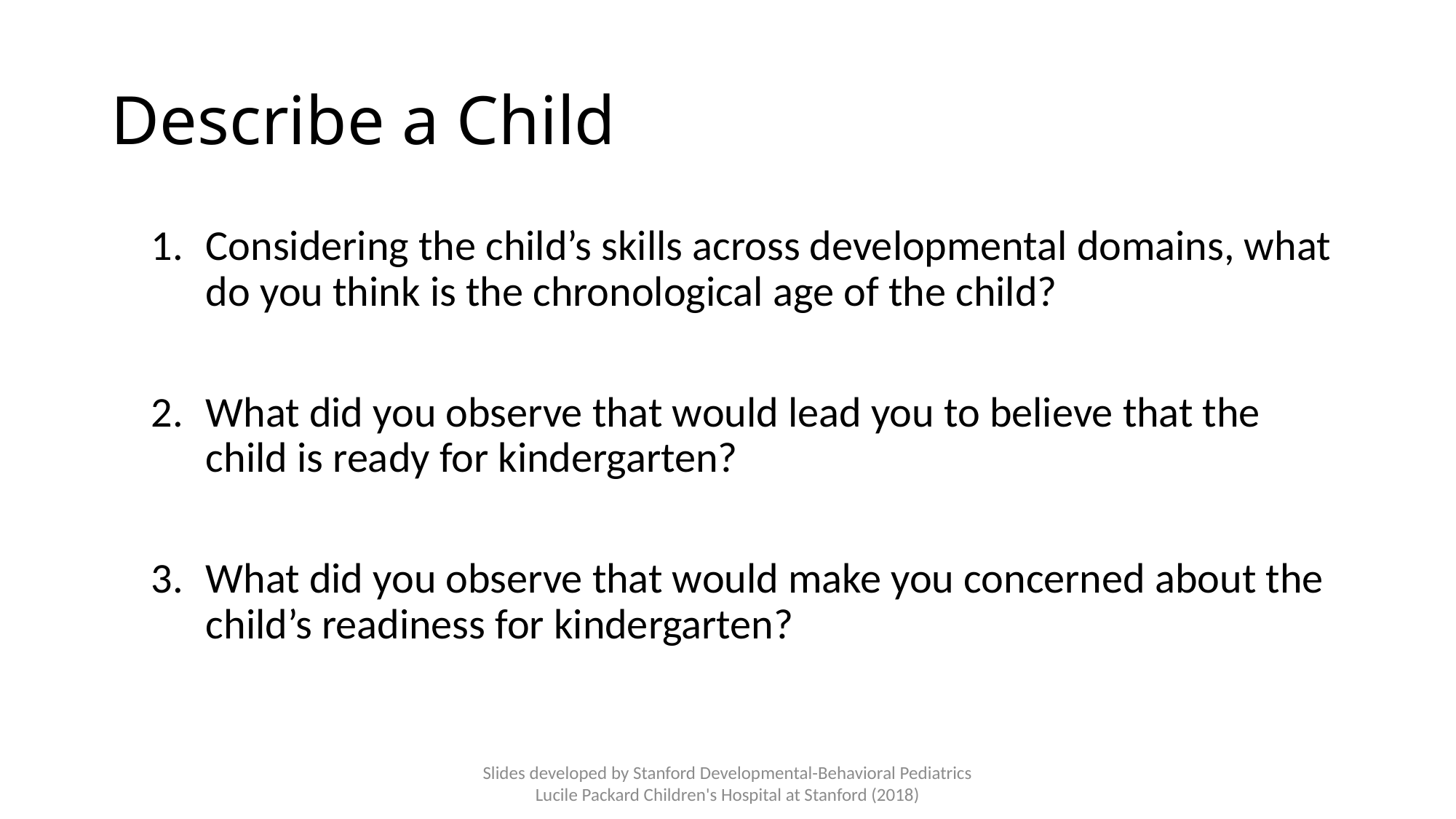

# Describe a Child
Considering the child’s skills across developmental domains, what do you think is the chronological age of the child?
What did you observe that would lead you to believe that the child is ready for kindergarten?
What did you observe that would make you concerned about the child’s readiness for kindergarten?
Slides developed by Stanford Developmental-Behavioral Pediatrics Lucile Packard Children's Hospital at Stanford (2018)

## Slide 13
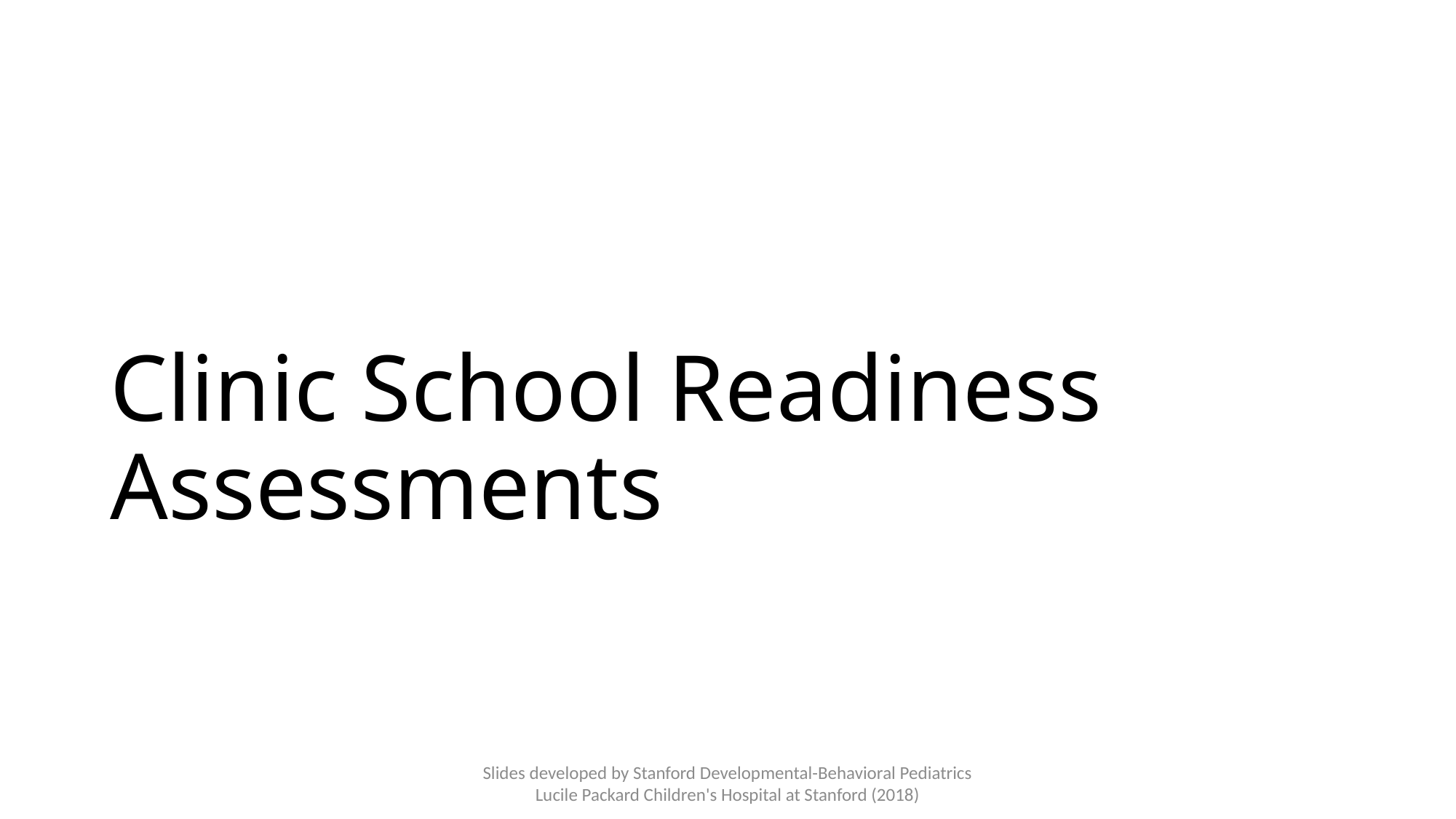

# Clinic School Readiness Assessments
Slides developed by Stanford Developmental-Behavioral Pediatrics Lucile Packard Children's Hospital at Stanford (2018)

## Slide 14
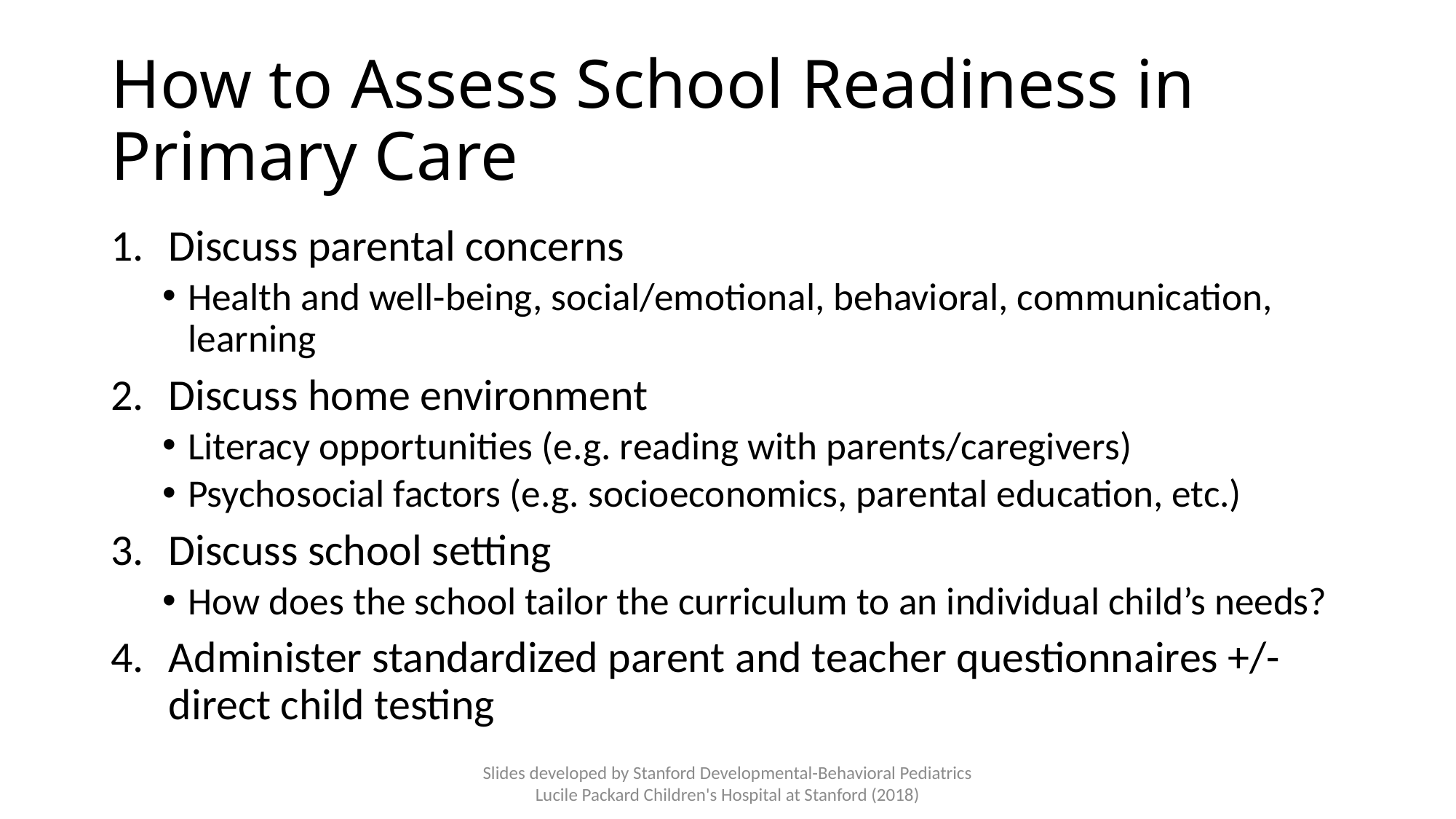

# How to Assess School Readiness in Primary Care
Discuss parental concerns
Health and well-being, social/emotional, behavioral, communication, learning
Discuss home environment
Literacy opportunities (e.g. reading with parents/caregivers)
Psychosocial factors (e.g. socioeconomics, parental education, etc.)
Discuss school setting
How does the school tailor the curriculum to an individual child’s needs?
Administer standardized parent and teacher questionnaires +/- direct child testing
Slides developed by Stanford Developmental-Behavioral Pediatrics Lucile Packard Children's Hospital at Stanford (2018)

## Slide 15
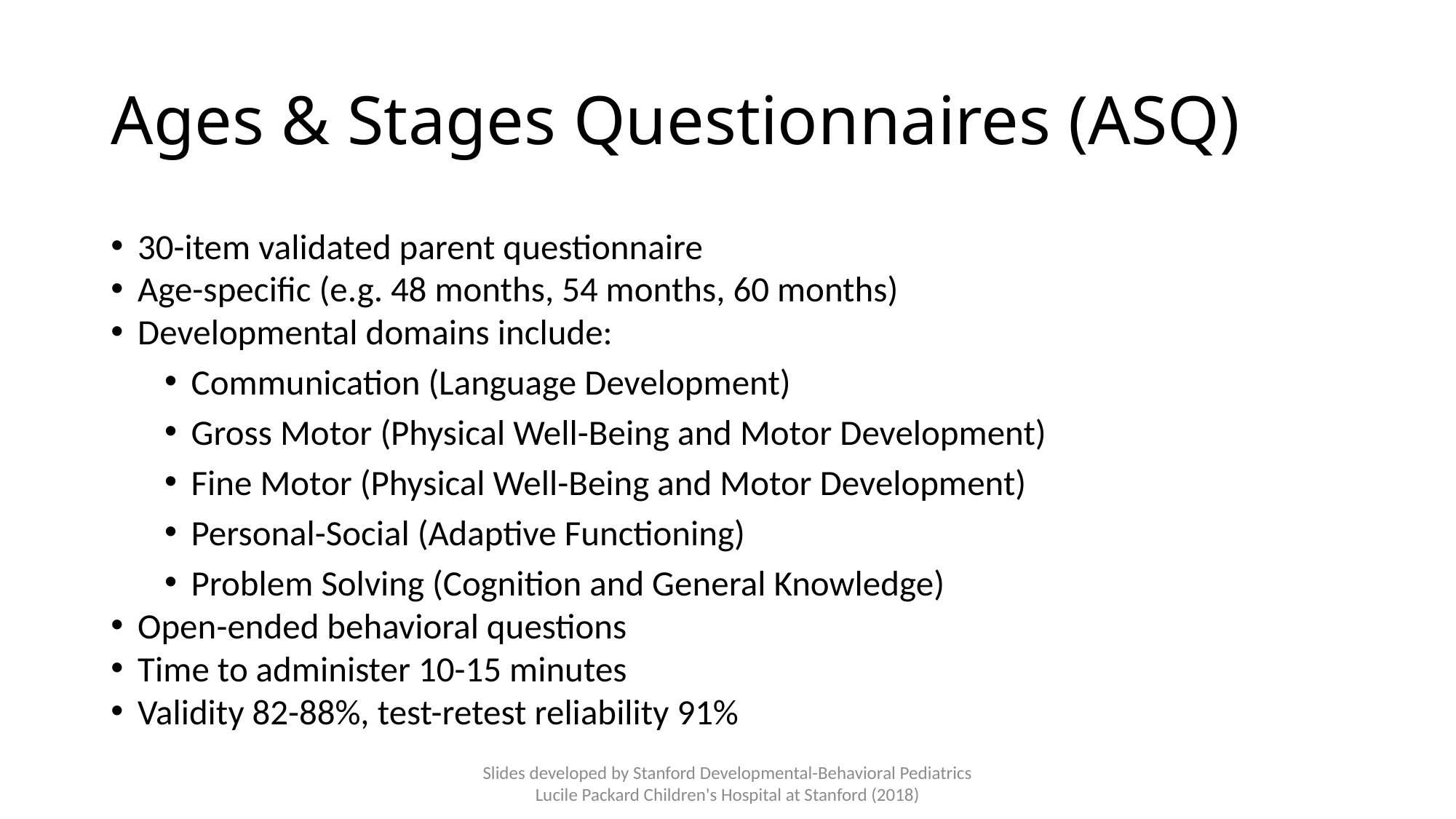

# Ages & Stages Questionnaires (ASQ)
30-item validated parent questionnaire
Age-specific (e.g. 48 months, 54 months, 60 months)
Developmental domains include:
Communication (Language Development)
Gross Motor (Physical Well-Being and Motor Development)
Fine Motor (Physical Well-Being and Motor Development)
Personal-Social (Adaptive Functioning)
Problem Solving (Cognition and General Knowledge)
Open-ended behavioral questions
Time to administer 10-15 minutes
Validity 82-88%, test-retest reliability 91%
Slides developed by Stanford Developmental-Behavioral Pediatrics Lucile Packard Children's Hospital at Stanford (2018)

## Slide 16
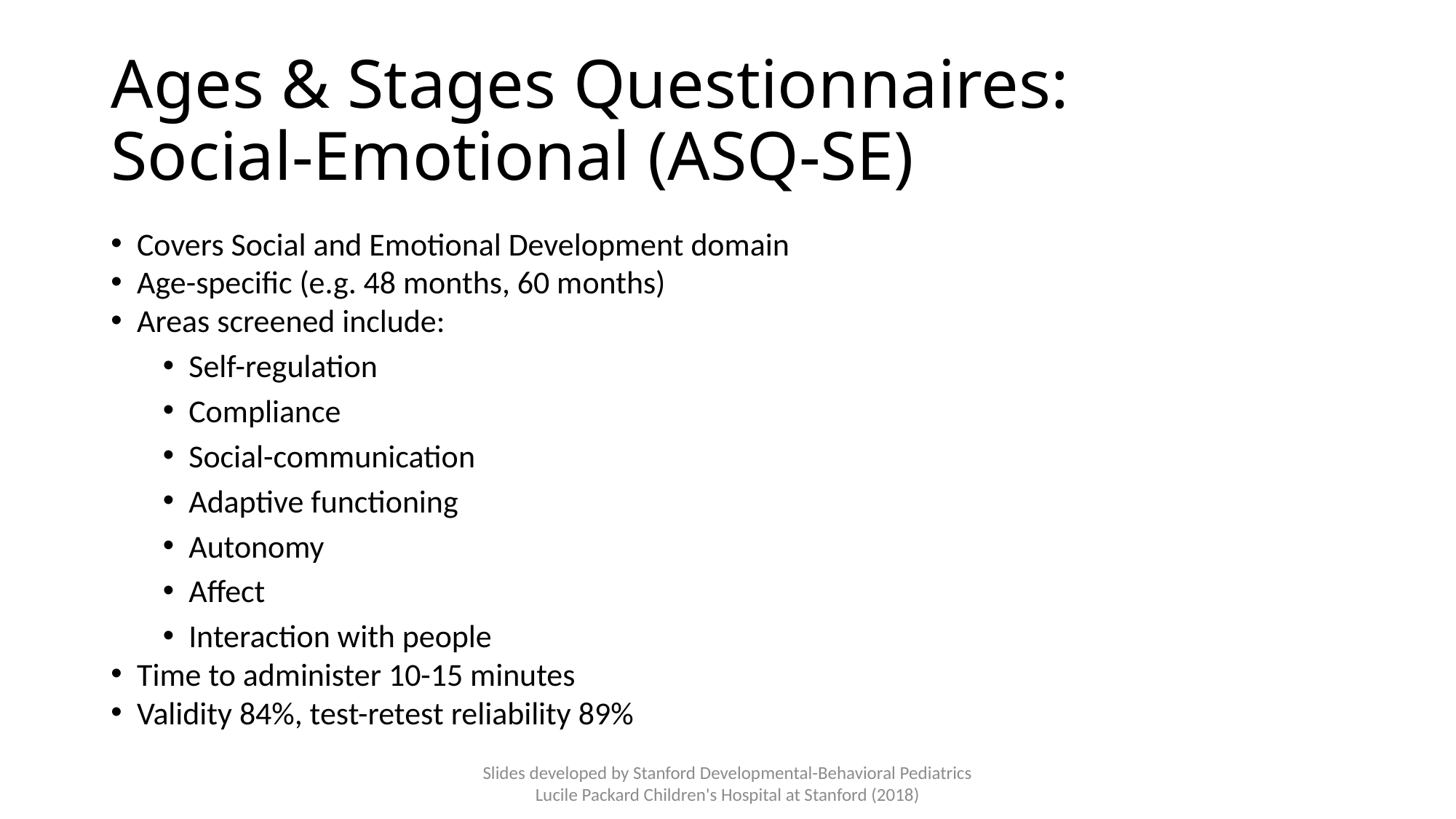

# Ages & Stages Questionnaires: Social-Emotional (ASQ-SE)
Covers Social and Emotional Development domain
Age-specific (e.g. 48 months, 60 months)
Areas screened include:
Self-regulation
Compliance
Social-communication
Adaptive functioning
Autonomy
Affect
Interaction with people
Time to administer 10-15 minutes
Validity 84%, test-retest reliability 89%
Slides developed by Stanford Developmental-Behavioral Pediatrics Lucile Packard Children's Hospital at Stanford (2018)

## Slide 17
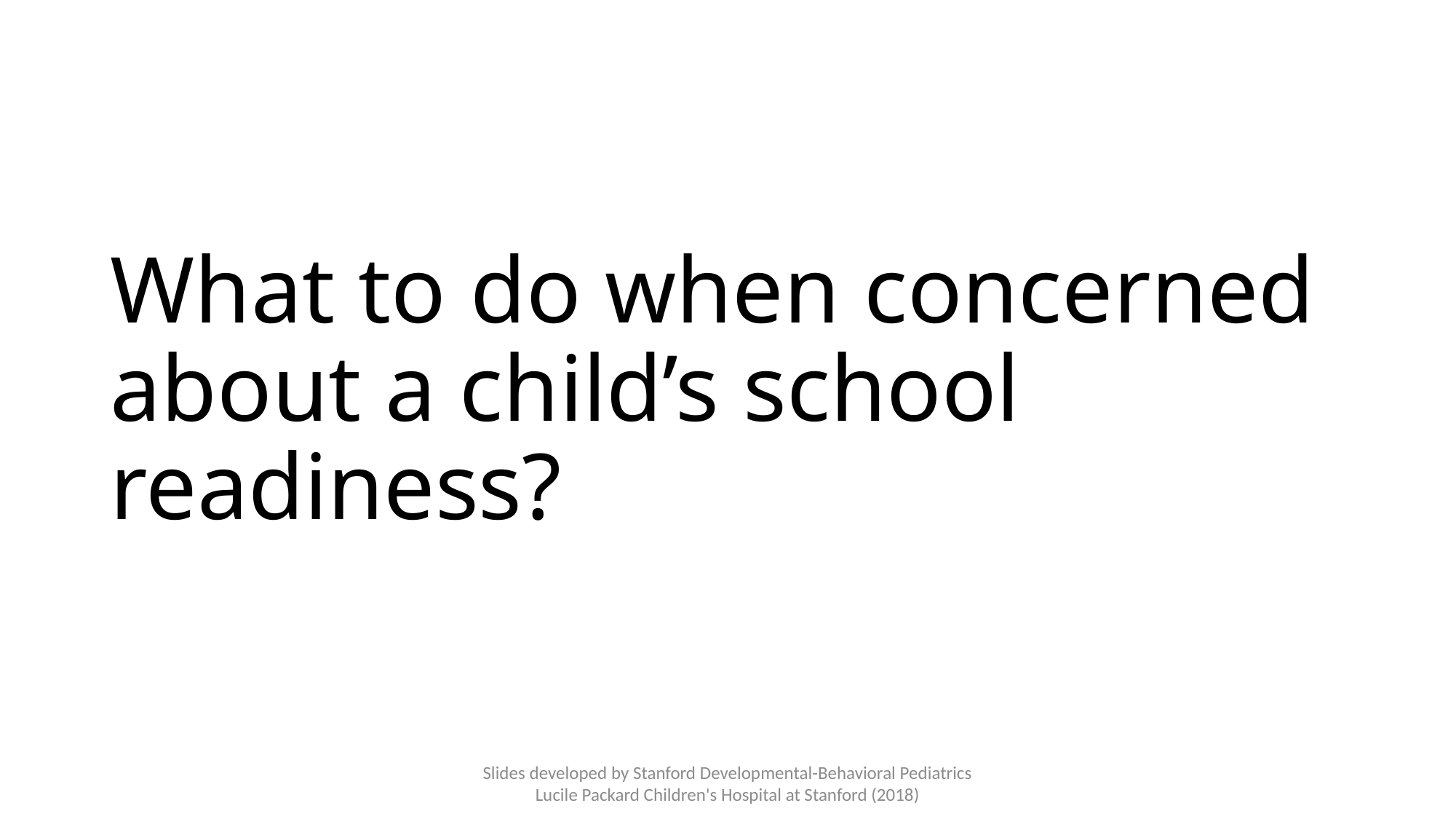

# What to do when concerned about a child’s school readiness?
Slides developed by Stanford Developmental-Behavioral Pediatrics Lucile Packard Children's Hospital at Stanford (2018)

## Slide 18
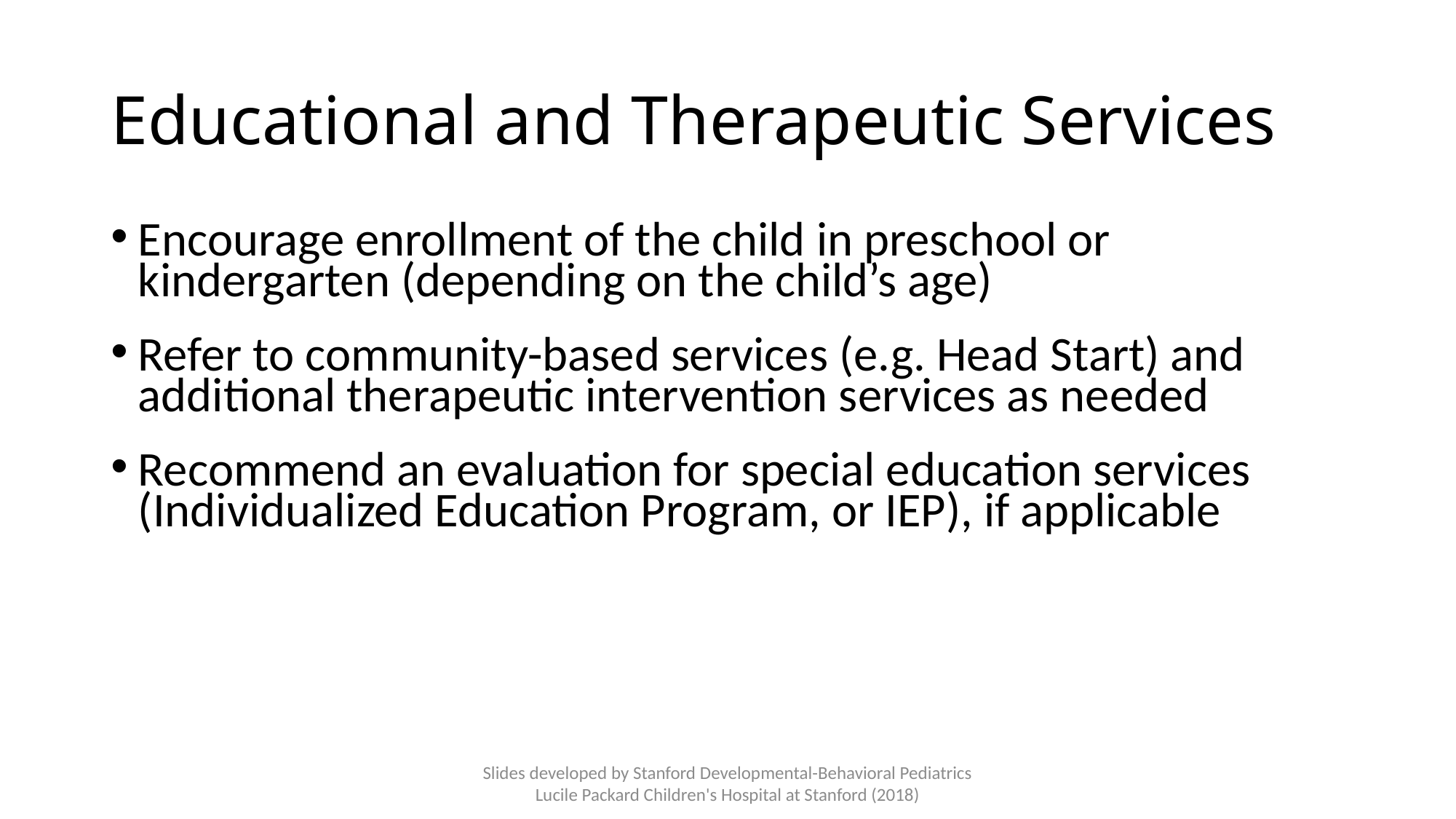

# Educational and Therapeutic Services
Encourage enrollment of the child in preschool or kindergarten (depending on the child’s age)
Refer to community-based services (e.g. Head Start) and additional therapeutic intervention services as needed
Recommend an evaluation for special education services (Individualized Education Program, or IEP), if applicable
Slides developed by Stanford Developmental-Behavioral Pediatrics Lucile Packard Children's Hospital at Stanford (2018)

## Slide 19
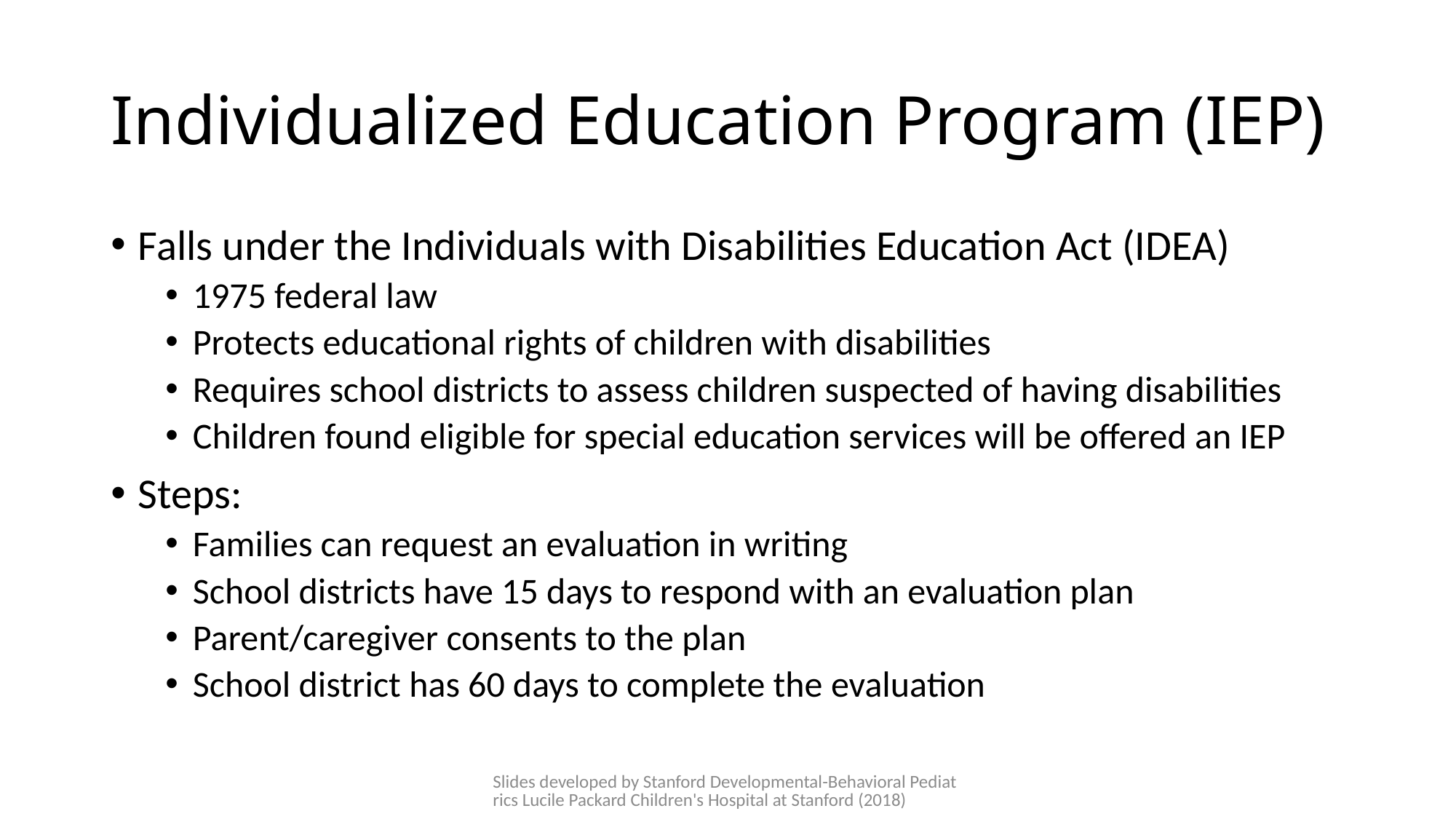

# Individualized Education Program (IEP)
Falls under the Individuals with Disabilities Education Act (IDEA)
1975 federal law
Protects educational rights of children with disabilities
Requires school districts to assess children suspected of having disabilities
Children found eligible for special education services will be offered an IEP
Steps:
Families can request an evaluation in writing
School districts have 15 days to respond with an evaluation plan
Parent/caregiver consents to the plan
School district has 60 days to complete the evaluation
Slides developed by Stanford Developmental-Behavioral Pediatrics Lucile Packard Children's Hospital at Stanford (2018)

## Slide 20
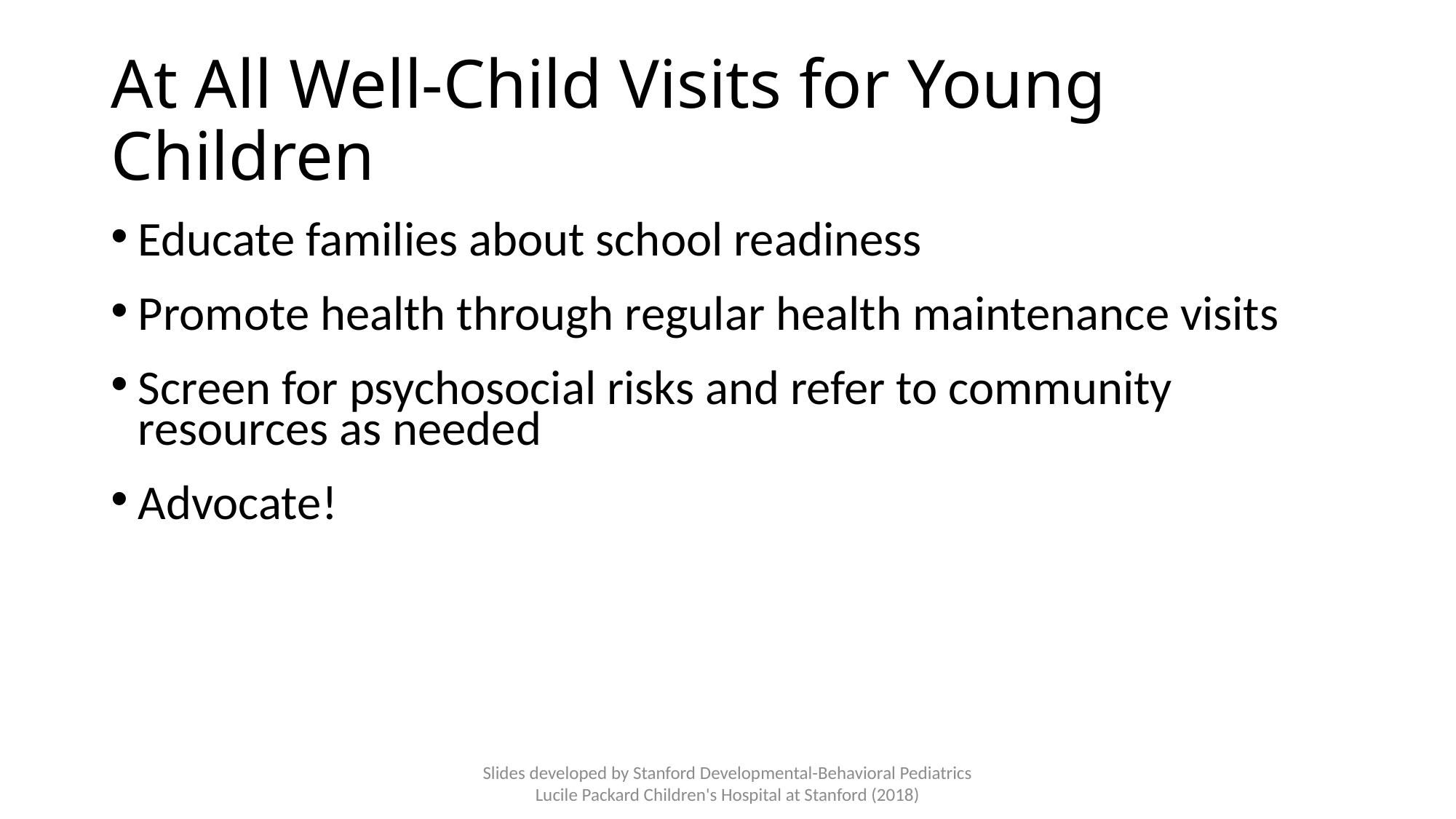

# At All Well-Child Visits for Young Children
Educate families about school readiness
Promote health through regular health maintenance visits
Screen for psychosocial risks and refer to community resources as needed
Advocate!
Slides developed by Stanford Developmental-Behavioral Pediatrics Lucile Packard Children's Hospital at Stanford (2018)

## Slide 21
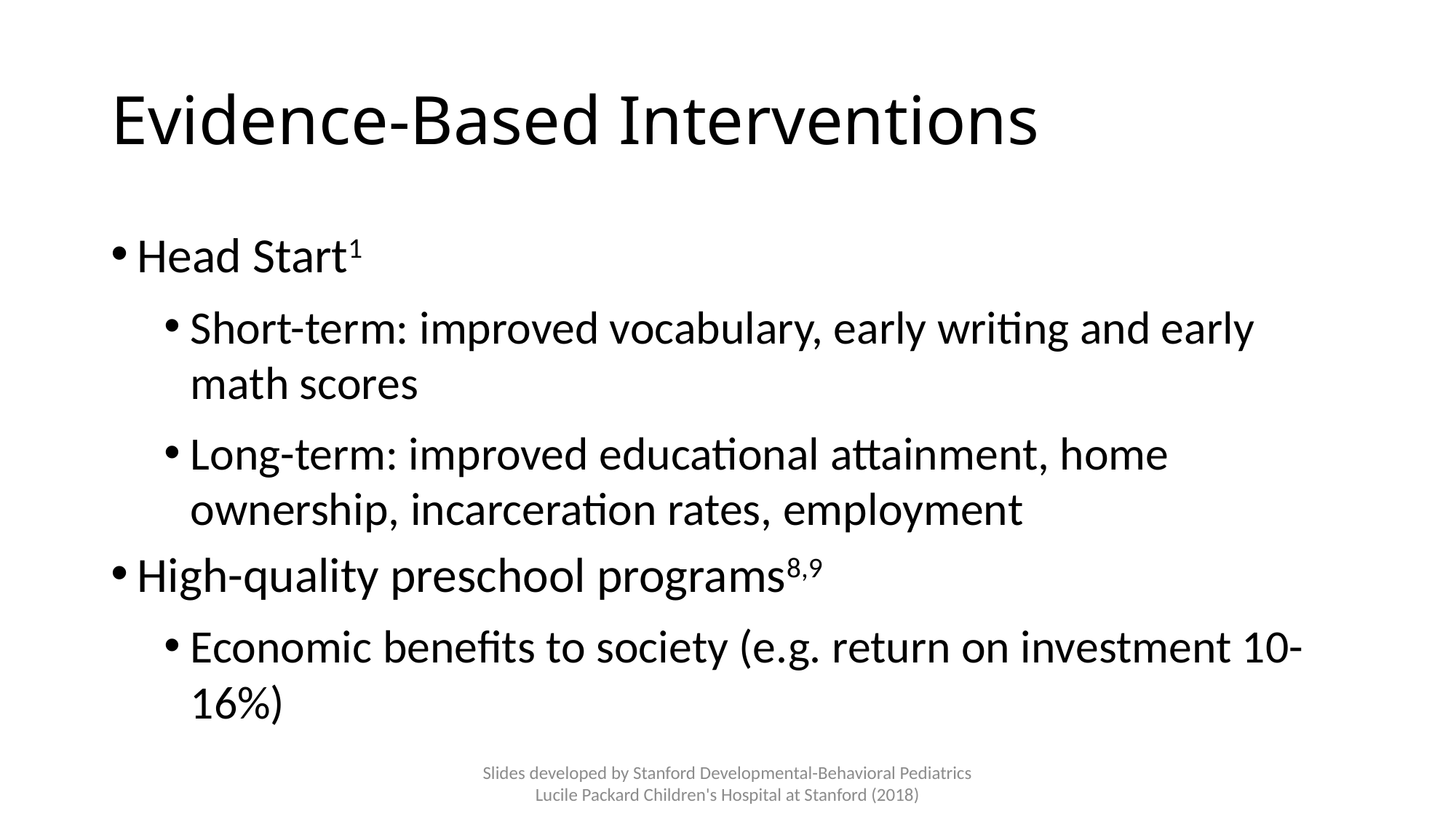

# Evidence-Based Interventions
Head Start1
Short-term: improved vocabulary, early writing and early math scores
Long-term: improved educational attainment, home ownership, incarceration rates, employment
High-quality preschool programs8,9
Economic benefits to society (e.g. return on investment 10-16%)
Slides developed by Stanford Developmental-Behavioral Pediatrics Lucile Packard Children's Hospital at Stanford (2018)

## Slide 22
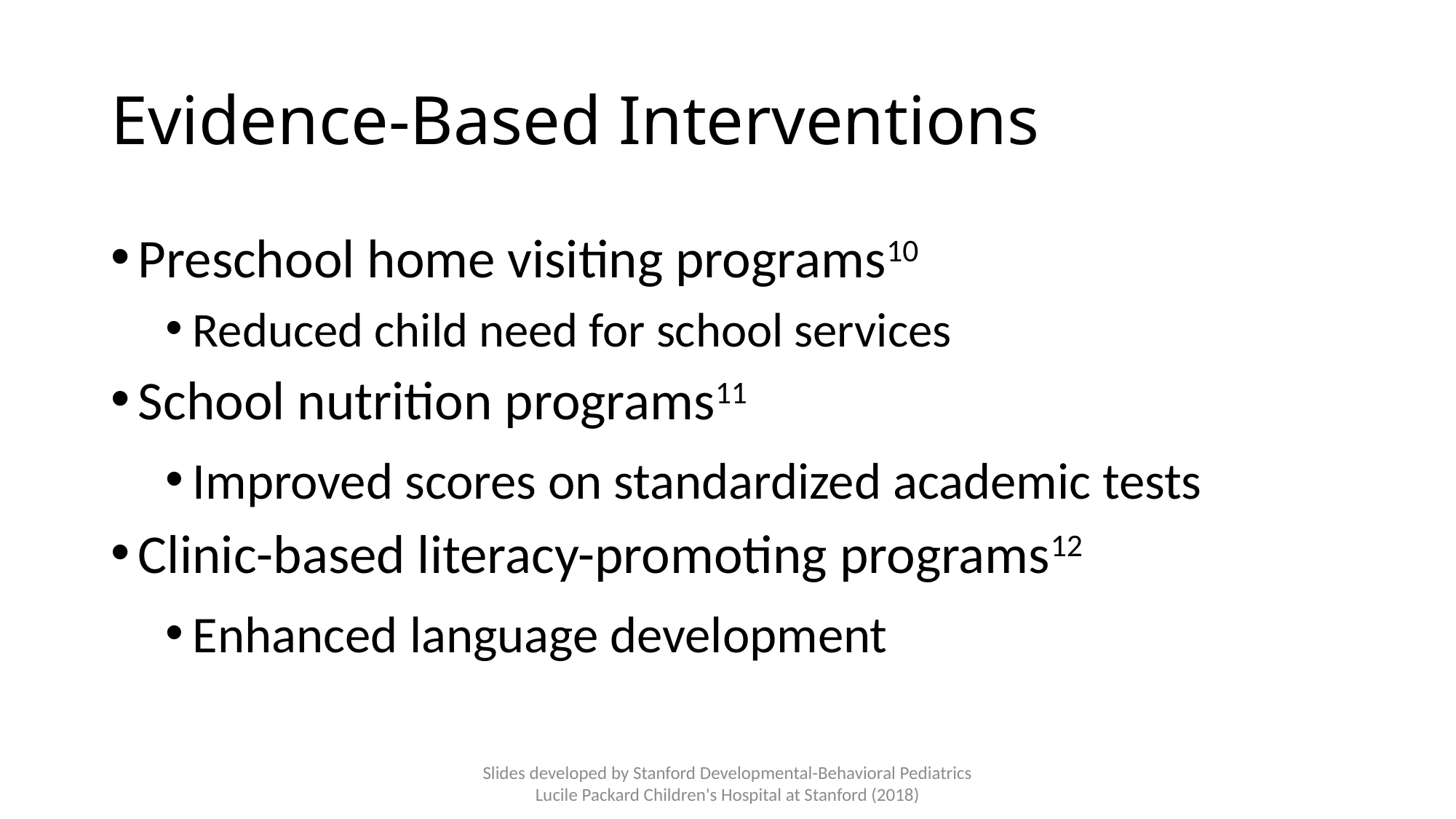

# Evidence-Based Interventions
Preschool home visiting programs10
Reduced child need for school services
School nutrition programs11
Improved scores on standardized academic tests
Clinic-based literacy-promoting programs12
Enhanced language development
Slides developed by Stanford Developmental-Behavioral Pediatrics Lucile Packard Children's Hospital at Stanford (2018)

## Slide 23
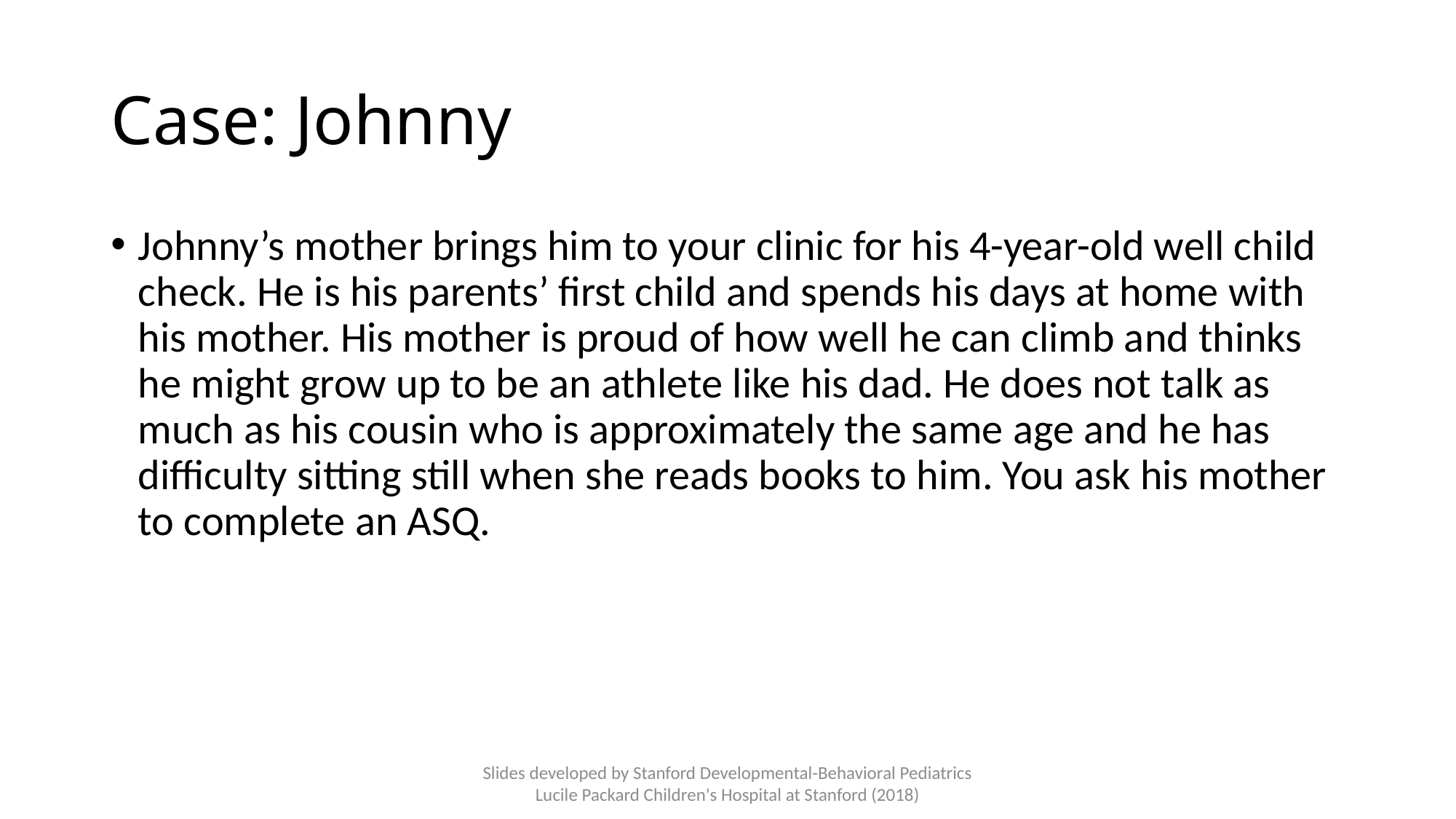

# Case: Johnny
Johnny’s mother brings him to your clinic for his 4-year-old well child check. He is his parents’ first child and spends his days at home with his mother. His mother is proud of how well he can climb and thinks he might grow up to be an athlete like his dad. He does not talk as much as his cousin who is approximately the same age and he has difficulty sitting still when she reads books to him. You ask his mother to complete an ASQ.
Slides developed by Stanford Developmental-Behavioral Pediatrics Lucile Packard Children's Hospital at Stanford (2018)

## Slide 24
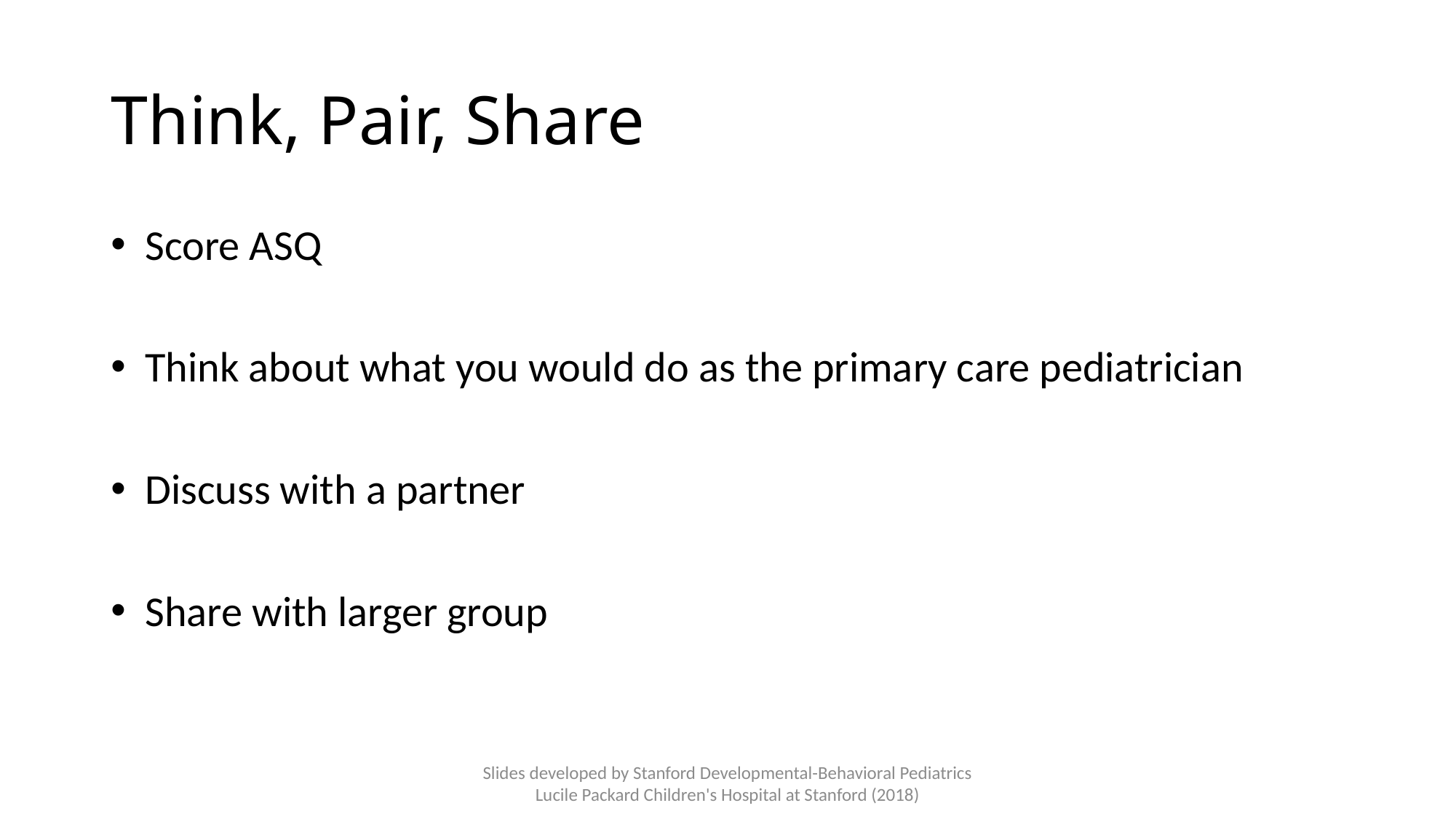

# Think, Pair, Share
Score ASQ
Think about what you would do as the primary care pediatrician
Discuss with a partner
Share with larger group
Slides developed by Stanford Developmental-Behavioral Pediatrics Lucile Packard Children's Hospital at Stanford (2018)

## Slide 25
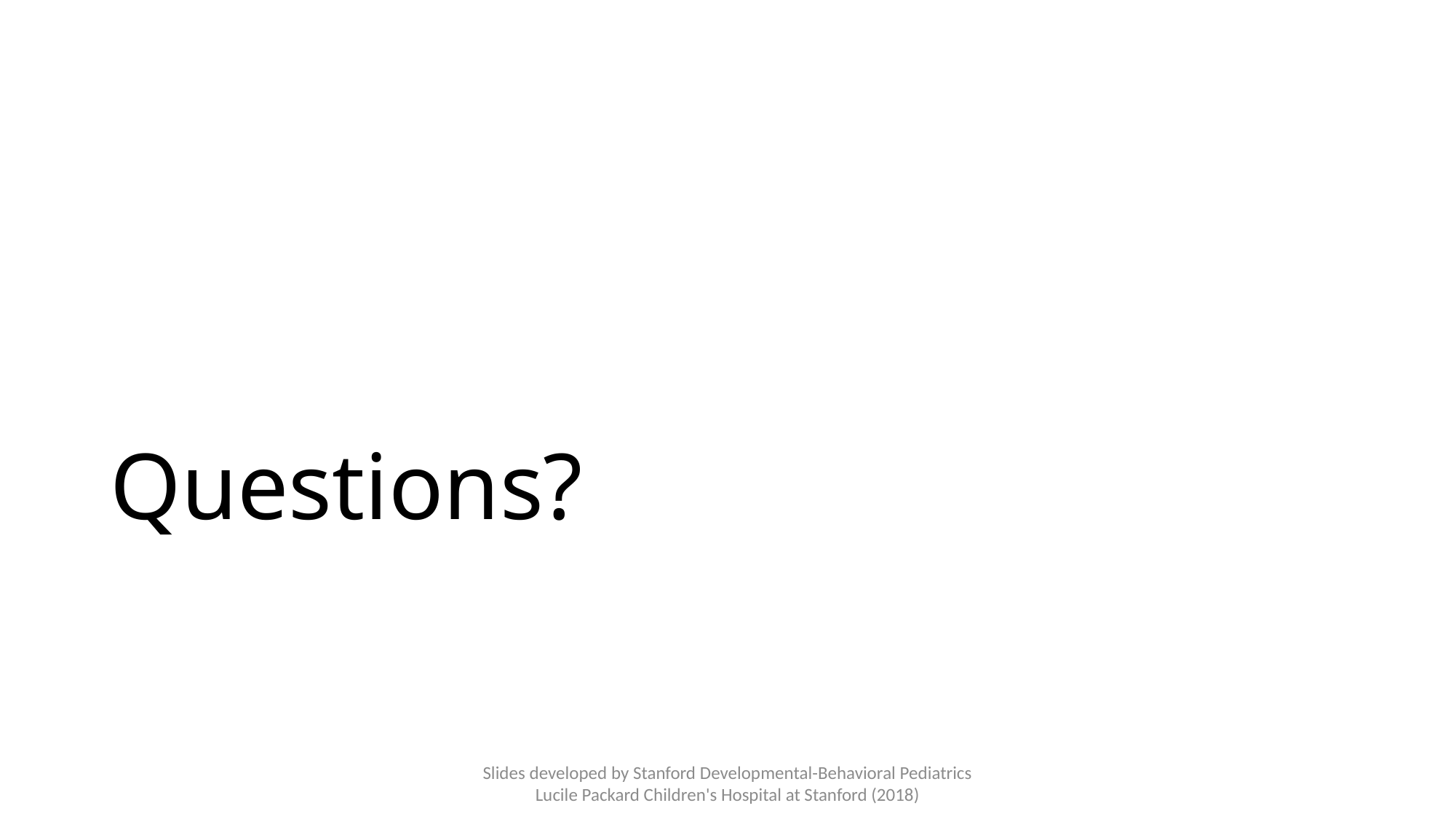

# Questions?
Slides developed by Stanford Developmental-Behavioral Pediatrics Lucile Packard Children's Hospital at Stanford (2018)

## Slide 26
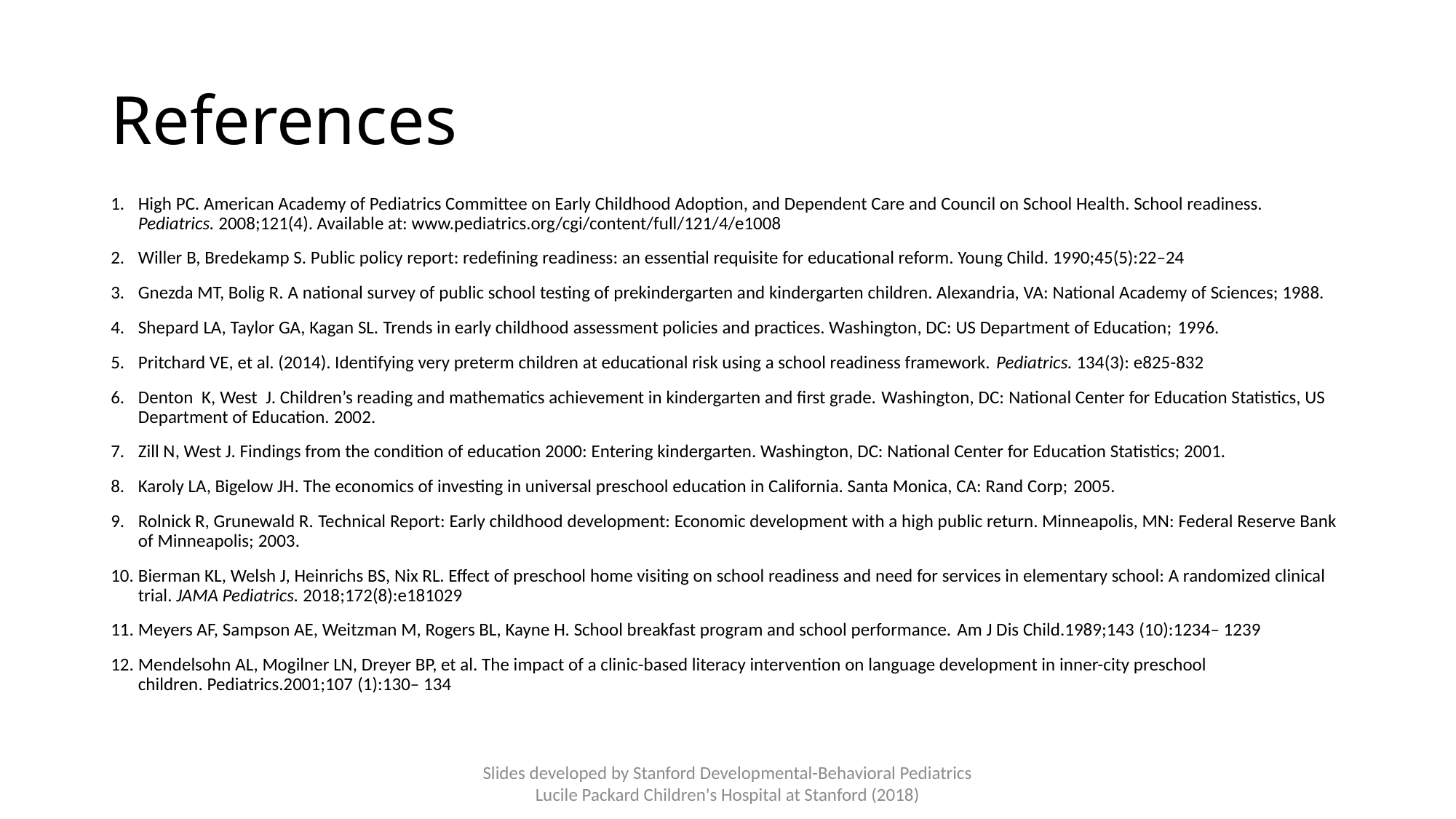

# References
High PC. American Academy of Pediatrics Committee on Early Childhood Adoption, and Dependent Care and Council on School Health. School readiness. Pediatrics. 2008;121(4). Available at: www.pediatrics.org/cgi/content/full/121/4/e1008
Willer B, Bredekamp S. Public policy report: redefining readiness: an essential requisite for educational reform. Young Child. 1990;45(5):22–24
Gnezda MT, Bolig R. A national survey of public school testing of prekindergarten and kindergarten children. Alexandria, VA: National Academy of Sciences; 1988.
Shepard LA, Taylor GA, Kagan SL. Trends in early childhood assessment policies and practices. Washington, DC: US Department of Education; 1996.
Pritchard VE, et al. (2014). Identifying very preterm children at educational risk using a school readiness framework. Pediatrics. 134(3): e825-832
Denton  K, West  J. Children’s reading and mathematics achievement in kindergarten and first grade. Washington, DC: National Center for Education Statistics, US Department of Education. 2002.
Zill N, West J. Findings from the condition of education 2000: Entering kindergarten. Washington, DC: National Center for Education Statistics; 2001.
Karoly LA, Bigelow JH. The economics of investing in universal preschool education in California. Santa Monica, CA: Rand Corp; 2005.
Rolnick R, Grunewald R. Technical Report: Early childhood development: Economic development with a high public return. Minneapolis, MN: Federal Reserve Bank of Minneapolis; 2003.
Bierman KL, Welsh J, Heinrichs BS, Nix RL. Effect of preschool home visiting on school readiness and need for services in elementary school: A randomized clinical trial. JAMA Pediatrics. 2018;172(8):e181029
Meyers AF, Sampson AE, Weitzman M, Rogers BL, Kayne H. School breakfast program and school performance. Am J Dis Child.1989;143 (10):1234– 1239
Mendelsohn AL, Mogilner LN, Dreyer BP, et al. The impact of a clinic-based literacy intervention on language development in inner-city preschool children. Pediatrics.2001;107 (1):130– 134
Slides developed by Stanford Developmental-Behavioral Pediatrics Lucile Packard Children's Hospital at Stanford (2018)
