# Supplementary material for: Development and Evaluation of a School Readiness Curriculum for Pediatrics Residents
Source: MedEdPORTAL. 2020 Sep 29;16:10976. doi: 10.15766/mep_2374-8265.10976 (PMC7526503; doi:10.15766/mep_2374-8265.10976)
Supplement: Supplementary file 1 — Preschool Observation Guide.docSchool Readiness Workshop.pptxDevelopmental Questionnaire.pdfPreintervention Survey.docxImmediate Postintervention Survey.docxDelayed Postintervention Survey.docx [file mep_2374-8265.10976-s001.zip › D. Preintervention Survey.docx]

**School Readiness Curriculum Preintervention Survey**

This survey is designed to assess your understanding of school readiness and your confidence in discussing school readiness with patients and families. Please answer honestly and to the best of your ability.

1. School readiness can best be described as…
   1. Child skill set needed for kindergarten success, consisting of pre-academic skills, social skills, language skills, motor skills, and behavior regulation.
   2. Multilevel characteristics needed for child kindergarten success, including child skill set, school adaptability, and family/community support.
   3. Child knowledge of all 26 letters and ability to count to 10.
   4. A standard score on the National School Readiness Exam ≥ 70.
2. How confident are you in your own ability to **discuss** school readiness with the family of a preschool-aged child?
   1. Not at all confident
   2. Slightly confident
   3. Moderately confident
   4. Very confident
   5. Extremely confident
3. How confident are you in your own ability to **address** **concerns** about school readiness in a preschool-aged child?
   1. Not at all confident
   2. Slightly confident
   3. Moderately confident
   4. Very confident
   5. Extremely confident
4. A 4-year-old child presents to your clinic in June. The child will turn 5 next month. You are concerned about the child’s lack of readiness for kindergarten in the fall. Which of the following recommendations is the **most** **appropriate** next step?
   1. Refer the child to Early Start for an evaluation for early intervention services.
   2. Advise the parent to defer kindergarten and enroll the child in preschool.
   3. Advise the parent to enroll the child in kindergarten.
   4. Send the child for an Intelligence Quotient (IQ) test.
5. Thinking back to the most recent Well Child Check (WCC) you had with a 4- or 5-year-old child, did **you** bring up the topic of school readiness?
   1. No
   2. Yes
   3. Not applicable (i.e. you have not seen a 4- or 5-year-old child for a WCC)
6. Thinking back to the most recent Well Child Check (WCC) you had with a 4- or 5-year-old child, did **the family** bring up the topic of school readiness?
   1. No
   2. Yes
   3. Not applicable (i.e. you have not seen a 4- or 5-year-old child for a WCC)
7. If you answered “Yes” to Questions 5 or 6, how prepared did you feel to answer the family’s questions?
   1. Not at all prepared
   2. Slightly prepared
   3. Moderately prepared
   4. Very prepared
   5. Extremely prepared
   6. Not applicable (i.e. the family did not have any questions)

**School Readiness Curriculum Pre-Intervention Survey Knowledge Items (correct answers in bold)**

1. School readiness can best be described as…

- 1. Child skill set needed for kindergarten success, consisting of pre-academic skills, social skills, language skills, motor skills, and behavior regulation.
  2. **Multilevel characteristics needed for child kindergarten success, including child skill set, school adaptability, and family/community support.**
  3. Child knowledge of all 26 letters and ability to count to 10.
  4. A standard score on the National School Readiness Exam ≥ 70.

4. A 4-year-old child presents to your clinic in June. The child will turn 5 next month. You are concerned about the child’s lack of readiness for kindergarten in the fall. Which of the following recommendations is the **most** **appropriate** next step?

- 1. Refer the child to Early Start for an evaluation for early intervention services.
  2. Advise the parent to defer kindergarten and enroll the child in preschool.
  3. **Advise the parent to enroll the child in kindergarten.**
  4. Send the child for an Intelligence Quotient (IQ) test.
