# Supplementary material for: Development and Evaluation of a School Readiness Curriculum for Pediatrics Residents
Source: MedEdPORTAL. 2020 Sep 29;16:10976. doi: 10.15766/mep_2374-8265.10976 (PMC7526503; doi:10.15766/mep_2374-8265.10976)
Supplement: Supplementary file 1 — Preschool Observation Guide.docSchool Readiness Workshop.pptxDevelopmental Questionnaire.pdfPreintervention Survey.docxImmediate Postintervention Survey.docxDelayed Postintervention Survey.docx [file mep_2374-8265.10976-s001.zip › E. Immediate Postintervention Survey.docx]

**School Readiness Curriculum Immediate-Postintervention Survey**

This survey is designed to assess your understanding of school readiness and your confidence in discussing school readiness with patients and families. The survey also invites feedback from the workshop. Please answer honestly and to the best of your ability.

1. School readiness can best be described as…
   1. Child skill set needed for kindergarten success, consisting of pre-academic skills, social skills, language skills, motor skills, and behavior regulation.
   2. Multilevel characteristics needed for child kindergarten success, including child skill set, school adaptability, and family/community support.
   3. Child knowledge of all 26 letters and ability to count to 10.
   4. A standard score on the National School Readiness Exam ≥ 70.
2. How confident are you in your own ability to **discuss** school readiness with the family of a preschool-aged child?
   1. Not at all confident
   2. Slightly confident
   3. Moderately confident
   4. Very confident
   5. Extremely confident
3. How confident are you in your own ability to **address** **concerns** about school readiness in a preschool-aged child?
   1. Not at all confident
   2. Slightly confident
   3. Moderately confident
   4. Very confident
   5. Extremely confident
4. A 4-year-old child presents to your clinic in June. The child will turn 5 next month. You are concerned about the child’s lack of readiness for kindergarten in the fall. Which of the following recommendations is the **most** **appropriate** next step?
   1. Refer the child to Early Start for an evaluation for early intervention services.
   2. Advise the parent to defer kindergarten and enroll the child in preschool.
   3. Advise the parent to enroll the child in kindergarten.
   4. Send the child for an Intelligence Quotient (IQ) test.

To what extent do you agree with the following statements?

1. This workshop enhanced my knowledge and/or skills to better prepare me to **discuss** school readiness with the family of a preschool-aged child. (Circle one)

Completely disagree Generally disagree Generally agree Completely agree

1. This workshop enhanced my knowledge and/or skills to better prepare me to **address concerns** about school readiness in preschool-aged children. (Circle one)

Completely disagree Generally disagree Generally agree Completely agree

1. Overall, how would you rate this workshop? (Circle one)

Poor Fair Good Very Good Excellent

1. What was most effective about this workshop? ____________________________________________________________________________________________

**School Readiness Curriculum Immediate-Postintervention Survey Knowledge Items (correct answers in bold)**

1. School readiness can best be described as…

- 1. Child skill set needed for kindergarten success, consisting of pre-academic skills, social skills, language skills, motor skills, and behavior regulation.
  2. **Multilevel characteristics needed for child kindergarten success, including child skill set, school adaptability, and family/community support.**
  3. Child knowledge of all 26 letters and ability to count to 10.
  4. A standard score on the National School Readiness Exam ≥ 70.

4. A 4-year-old child presents to your clinic in June. The child will turn 5 next month. You are concerned about the child’s lack of readiness for kindergarten in the fall. Which of the following recommendations is the **most** **appropriate** next step?

- 1. Refer the child to Early Start for an evaluation for early intervention services.
  2. Advise the parent to defer kindergarten and enroll the child in preschool.
  3. **Advise the parent to enroll the child in kindergarten.**
  4. Send the child for an Intelligence Quotient (IQ) test.
