# Supplementary material for: Development and Evaluation of a School Readiness Curriculum for Pediatrics Residents
Source: MedEdPORTAL. 2020 Sep 29;16:10976. doi: 10.15766/mep_2374-8265.10976 (PMC7526503; doi:10.15766/mep_2374-8265.10976)
Supplement: Supplementary file 1 — Preschool Observation Guide.docSchool Readiness Workshop.pptxDevelopmental Questionnaire.pdfPreintervention Survey.docxImmediate Postintervention Survey.docxDelayed Postintervention Survey.docx [file mep_2374-8265.10976-s001.zip › A. Preschool Observation Guide.doc]

### Stanford Developmental-Behavioral Pediatrics Rotation: A Guide to Preschool Observations^[1]^

This experience is designed to help you learn more about the normal developmental differences of young children of various ages as you watch them interact with each other and their teachers. You will be asked to observe the classroom environment, the staff and the children, as well as focus extensively on one particular child.

# General Guidelines to Being an Observer

Find a comfortable place to sit with a good view of most of the room.

Keep in the background so that the teachers and children can forget you are there.

If the children ask what you are doing, say something truthful but ambiguous, such as "writing.”

If the children ask for your help, direct them to a teacher whenever possible.

Try to keep any particular child you are observing from being overly conscious of your attention, by making regular glances to other places or children in the room.

# Objective vs. Subjective Records

When observing children, it is tempting to interpret their behavior in terms of "good" or "bad,” "pleasing" or "displeasing", etc. It is important to describe or record objectively what the child does or says without interpretation. Accurate records are more usable for later interpretation.

# A Subjective Record

Mary is playing with Jane. They are not playing well together because Jane keeps pestering Mary. They play only a short time before Mary goes to play alone. She does not like to play with Jane.

# An Objective Record

Mary and Jane are building a block house. Jane knocks over the house. Mary puts it up again. Jane knocks it over a second time. Mary says, "I'm not going to play anymore.” Mary goes to the doll corner, picks up a doll, and rocks it.

# Recording Your Observations

The attached forms are provided to help structure your observations and will be used as the basis for discussion in your later "debriefing" session with your faculty supervisor. Please note the grid at the bottom of page 5, entitled "Staff Comments.” This allows you to keep a running record of the positive or negative nature of comments made by staff to individual children or groups of children (by entering slash marks), and thus to get a sense of whether the environment provides overwhelmingly positive, a balance of positive and negative, or predominately negative feedback to the children.

#### Negative Comments

"Linda, don't do it that way.”

"Children, we're going to just sit here until you can learn to behave."

"Tim, you won't have any friends if you can't learn to share the toys."

#### Positive Comments

"Jenny, I like how nicely you cleared your place at the table."

"What a great job you all did listening to our story today!"

"Ben, I'm really impressed with how you shared that truck with Maria!"

Observations

Observer Name: _______________________

Time at beginning of observation: _________

Time at end of observation: ______________

# I. The Child

First Name:__________________________

Age: _______________________________

How long at school: ___________________

Number of siblings: ____________________

Birth Order: __________________________

Physical and Motor Development

How does the child compare in size and weight with the other children?

Compare this child with the other children in skills requiring the use of his/her body:

Note the way the child walks. Does the walk seem typical for this group of children, or is it different?

Note the way the child runs. Did you see the child skip, jump, or not?

Describe how the child goes up and down stairs, climbs on toys such as a jungle gym, and rides a tricycle or other wheeled toys.

Compare this child with other children in dexterity and small muscle coordination.

Describe the way the child works with small items such as puzzles, pegs, art materials, dressing dolls, scissors, etc.

Describe the child's skills in putting on his/her clothing, buttoning, zipping, washing and drying hands, and eating. Can the child tie shoes?

How much time does the child remain in activities which require moving around the room, verses activities which require sitting still? How much is this behavior influenced by the program?

Social and Emotional Development

Behavior with adults

Did you see the child take leave of the parent in the morning? Describe:

Does the child seek the companionship of adults in preference to children? Does the child have an attachment to a particular adult?

How does the child seek adult approval and help?

Does the child accept adults' suggestions and redirection? What is the child's response to limits set by adults?

Behavior with other children

How does the child enter group play, e.g., immediately joins in or stands and observes first? Describe the child's behavior in group play (e.g., leader, follower, both?)

Does the child seem to prefer to play alone? Describe and compare the child's behavior with that of the other children. During what activities does the child prefer to play alone?

Does the child tend to play with a particular friend(s)? Describe.

When this child and another do not agree, what does the child do? (e.g., give in, compromise, yell for help, etc.)

Expression of feelings

How does the child show feelings of happiness and unhappiness? What other feelings did you see the child express?

How does the child show affection for another child or adult?

Did you see the child attempt to hurt another child either verbally or physically? What seemed to be the provocation? How was the situation handled?

Did you see the child respond to frustration, such as having to wait for a toy? What did the child do?

Cognitive Development

Compare this child's level of development with that of the other children in the following skill areas:

About how long is the child's attention span in different activities?

Is the child generally alert and able to solve tasks and problems?

Describe the child's memory skills: e.g., ability to remember people, directions, stories, songs and related past experiences.

How would you describe the language development of this child: articulation, fluidity, volume, vocabulary, sentence formation, and creative language? Write down some speech samples.

Did you observe the child's knowledge of any of the following: colors, size, numbers, time, and letters? Any other concepts?

Play

Does this child use creative materials (e.g., clay, paint) and how? Compare with other children.

Did the child participate in any musical activities, or seem interested in books or stories? Describe and compare with other children.

Did you see the child use dramatic play (pretending to be something or someone else)? Describe the play.

#### Overall Impressions of the Child

What do you see as this child's strengths developmentally?

If the child is 4-5 years old, what observed skills suggest that s/he will soon be ready for kindergarten?

Are there skills that are less well-developed relative to those of the child's classmates?

What about this child makes the most lasting impression on you?

II. The Environment

What do you notice about the layout of the rooms, the equipment, and materials that are conducive to working well with a group of young children?

Anything you would add, change or question in the environment?

Describe the outdoor playground or indoor gross motor activity areas.

What are the rules that maximize safety during gross motor play?

III. The Staff

Which teachers seem particularly effective? What do they do or not do with children, which leads you to your conclusion?

| Give examples you observe of the staff doing the following: | How did the child(ren) respond? |
| --- | --- |
| Praising/reinforcing a child's behavior |  |
| Redirecting (e.g. to another activity) |  |
| Disciplining |  |
| Comforting |  |
| Motivating, teaching problem solving |  |
| Being honest/building trust |  |

List examples of some topics of casual conversation observed between children and staff:

Staff Comments (record of positive or negative comments made by staff to an individual child or groups of children)

|  | Positive Comments | Negative Comments |
| --- | --- | --- |
| Individual child |  |  |
| Group of children |  |  |

1. Document developed and updated 20200308 by Developmental-Behavioral Pediatrics Lucile Packard Children's Hospital at Stanford. Document is an adaptation of unpublished form used by S. Claire Lorch and Barbara W. Boat at University of North Carolina, Chapel Hill, NC (see related publication Sharp MC, Lorch SC. A community outreach training program for pediatric residents and medical students. Journal of Medical Education. 1988 Apr;63(4):316-322. doi: 10.1097/00001888-198804000-00008)
